# *Bidens pilosa* L. (Asteraceae): Botanical Properties, Traditional Uses, Phytochemistry, and Pharmacology

**DOI:** 10.1155/2013/340215

**Published:** 2013-07-01

**Authors:** Arlene P. Bartolome, Irene M. Villaseñor, Wen-Chin Yang

**Affiliations:** ^1^Institute of Chemistry, University of the Philippines, Diliman, Quezon City 1101, Philippines; ^2^Agricultural Biotechnology Research Center, Academia Sinica, Taipei 115, Taiwan; ^3^Institute of Pharmacology, Yang-Ming University, Taipei 112, Taiwan; ^4^Department of Life Sciences, National Chung-Hsing University, Taichung 402, Taiwan; ^5^Institute of Zoology, National Taiwan University, Taipei 106, Taiwan; ^6^Department of Aquaculture, National Taiwan Ocean University, Keelung 20224, Taiwan

## Abstract

There are 230 to 240 known *Bidens* species. Among them, *Bidens pilosa* is a representative perennial herb, globally distributed across temperate and tropical regions. *B. pilosa* has been traditionally used in foods and medicines without obvious adverse effects. Despite significant progress in phytochemical and biological analyses of *B. pilosa* over the past few years, comprehensive and critical reviews of this plant are anachronistic or relatively limited in scope. The present review aims to summarize up-to-date information on the phytochemistry, pharmacology, and toxicology of *B. pilosa* from the literature. In addition to botanical studies and records of the traditional use of *B. pilosa* in over 40 diseases, scientific studies investigating the potential medicinal uses of this species and its constituent phytochemicals for a variety of disorders are presented and discussed. The structure, bioactivity, and likely mechanisms of action of *B. pilosa* and its phytochemicals are emphasized. Although some progress has been made, further rigorous efforts are required to investigate the individual compounds isolated from *B. pilosa* to understand and validate its traditional uses and develop clinical applications. The present review provides preliminary information and gives guidance for further basic and clinical research into this plant.

## 1. Introduction

The United Nations World Health Organization estimates that as many as 5.6 billion people, 80% of the world population, utilize herbal medicine for primary health care [1]. Plants have formed the foundation of complicated traditional medicine systems for thousands of years. Medicinal herbs are applied to treat a wide range of disease categories. The first written documentation of the use of medicinal herbs dates from the 26th century BCE in Mesopotamia, and the first record of the use of medicinal herbs by the Egyptians and Greeks dates from 18th century BCE and the 5th century BCE, respectively. Starting around the 11th century BCE, the Chinese and Indians started to develop herbal medicine systems, Chinese herbal medicine, and Ayurvedic medicine, respectively, that continue to be widely practiced today [2]. Therefore, since antiquity, medicinal herbs have played a prominent role in human health.

*B. pilosa* is an easy-to-grow herb that is widely distributed all over the world. It is considered to be a rich source of food and medicine for humans and animals [3, 4]. There is increasing global interest in the use of *B. pilosa* as shown by the many studies conducted on the plant in recent years. The folkloric use of *B. pilosa* has been recorded in America, Africa, Asia, and Oceania [5]. To explore the potential clinical application of *B. pilosa*, it is important to link its traditional use with rigorous evidence-based scientific study. The present review focuses on recent studies on the botany, traditional usage, phytochemistry, pharmacology, and toxicology of *B. pilosa*. The information provided here highlights the possible usefulness of *B. pilosa* and its isolated compounds and offers insights into possible future research directions. Studies of *B. pilosa* are divided into three groups: (1) the botany, ethnomedical uses, plant chemistry, pharmacology, and biosafety of *B. pilosa*; (2) scientific studies that validate the ethnomedical uses of *B. pilosa*; and (3) the therapeutic and future research potential of *B. pilosa*. 

### 1.1. Botany

*B. pilosa* was first collected and named by Carl Linnaeus in 1753 [3]. Taxonomically, it is assigned to the *Bidens *genus (Asteraceae) as shown in Table 1. This genus is estimated to include 230 to 240 species worldwide [3, 4]. *B. pilosa* has several varieties such as *B. pilosa *var. *radiata*, var. *minor*, var. *pilosa,* and var. *bisetosa*. Alongside examination of morphological traits, authentication of *B. pilosa *can be aided by chemotaxonomy and molecular characterization [7]. 

**Figure 1:**
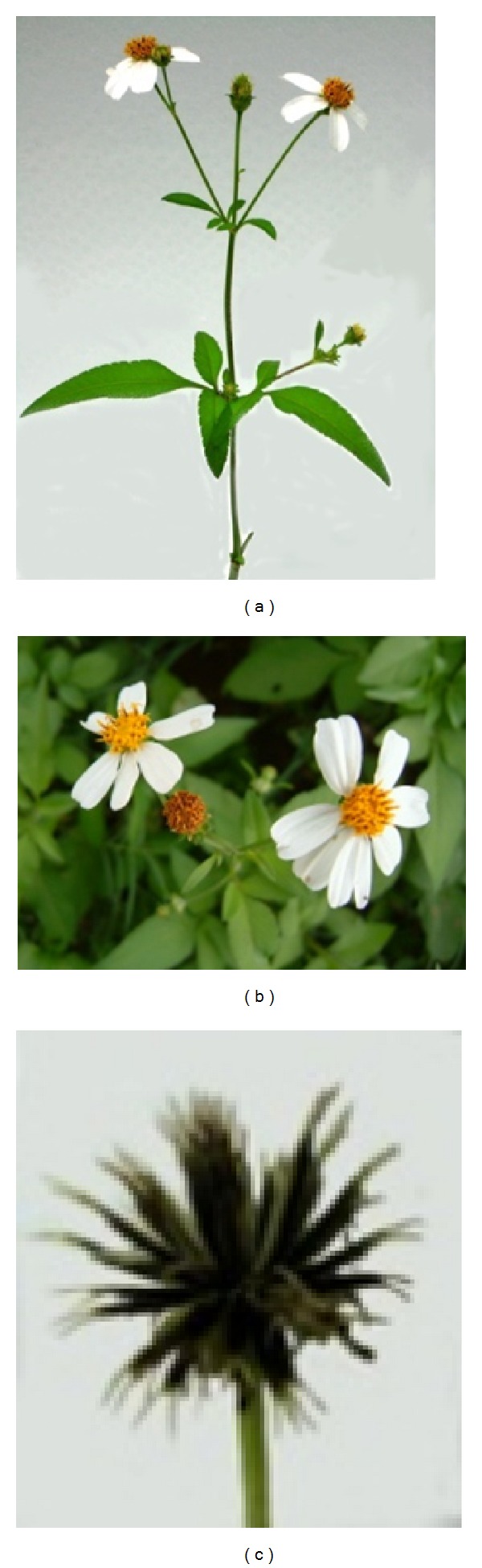
*B. pilosa *(a) and its flowers (b) and achenes (c).

*B. pilosa* is an erect, perennial herb widely distributed across temperate and tropical regions. *B. pilosa* is either glabrous or hairy, with green opposite leaves that are serrate, lobed, or dissected. It has white or yellow flowers, and long narrow ribbed black achenes (seeds). It grows to an average height of 60 cm and a maximum of 150 cm in favorable environments [8] (Figure 1). *B. pilosa *prefers full sun and moderately dry soil. However, it can grow in arid and barren land from low to high elevations. With the advantage of being fast-growing, in the 1970s, the Food and Agricultural Organization actively promoted the cultivation of *B. pilosa* in Africa [9]. *B. pilosa* propagates via seeds. A single plant can produce 3000–6000 seeds. Dry mature seeds from *B. pilosa* can be germinated in 3 to 4 days in moist soil or after being soaking in water. Seeds are viable for at least 3 years [10]. Minimal agricultural techniques are required for *B. pilosa* cultivation. Due to its invasive tendencies, *B. pilosa *is generally considered to be a weed [11].

*B. pilosa *is thought to have originated in South America and subsequently spread all over the world [12]. *Bidens *species and their varieties bear vernacular names based on their characteristics. For example, *Bidens* species are known by such names as Spanish needles, beggar's ticks, devil's needles, cobbler's pegs, broom stick, pitchforks, and farmers' friends in English and some other languages because of their sticky achenes [8] and are sometimes known as *xian feng cao* (“all bountiful grass”) in Chinese because of their prosperous growth.

### 1.2. Traditional Uses

*B. pilosa *is used as an herb and as an ingredient in teas or herbal medicines. Its shoots and leaves, dried or fresh, are utilized in sauces and teas [13, 14]. In the 1970s, the United Nations Food and Agriculture Organization (FAO) promoted the cultivation of *B. pilosa* in Africa because it is easy to grow, edible, palatable, and safe [15]. The nutritional value of *B. pilosa* is shown in Table 2. 

All parts of *B. pilosa* plant, the whole plant, the aerial parts (leaves, flowers, seeds, and stems), and/or the roots, fresh or dried, are used as ingredients in folk medicines. It is frequently prepared as a dry powder, decoction, maceration or tincture [16]. Generally, this plant is applied as dry powder or tincture when used externally, and as a powder, maceration, or decoction when used as an internal remedy [14]. 

As summarized in Table 3, *B. pilosa*, either as a whole plant or different parts, has been reported to be useful in the treatment of more than 40 disorders such as inflammation, immunological disorders, digestive disorders, infectious diseases, cancers, metabolic syndrome, wounds, and many others [17–20]. *B. pilosa* is usually ingested; however, it can also be utilized externally. For instance, fresh *B. pilosa* is used to treat snake bites and wounds [21], and in Trinidad and Tobago the aqueous solution of the leaves of *B. pilosa* is used to bathe babies and children [22]. 

*B. pilosa* is sometimes used alone; it is also used as an ingredient in medicinal mixtures together with other medicinal plants such as *Aloe vera*, *Plectranthus mollis*, *Valeriana officinalis*, and *Cissus sicyoides* among others [23–26]. Whether the commonly used mixtures of *B. pilosa,* or the mixtures of the compounds found in *B. pilosa* afford synergistic effects is not yet clear and needs to be verified by further studies. However, one study has suggested that *B. pilosa *varieties share similar phytochemical compositions and may be substituted for each other [7].

## 2. Phytochemicals

Interest in basic research and application of *B. pilosa *has increased since its first identification in 1753. This is mainly due to its wide application in medicines, foods, and drinks. Around 116 publications have documented the exploitation and medical use of *B. pilosa*. To date, 201 compounds comprising 70 aliphatics, 60 flavonoids, 25 terpenoids, 19 phenylpropanoids, 13 aromatics, 8 porphyrins, and 6 other compounds, have been identified from this plant as compiled previously [27]. The structures of these compounds are presented in Tables 4, 5, 6, 7, 8, 9, and 10, respectively. However, the association between *B. pilosa *phytochemicals and their bioactivities is not yet fully established and should become a future research focus. In the present review, we explore possible associations (Table 11), describe the importance of the known compounds in relation to their biological activity and discuss their likely mechanisms of action (Table 3). Compelling evidence suggests that the various diverse bioactivities reported for *B. pilosa* reflect its phytochemical complexity.

*B. pilosa* is an extraordinary source of phytochemicals, particularly flavonoids and polyynes. Plant flavonoids are commonly reported to possess anticancer, antiinflammatory, antioxidant, and other bioactivities. However, the bioactivities of only seven of the 60 flavonoids present in *B. pilosa* have been studied. The bioactivities of the remaining 53 flavonoids are poorly understood and deserve further investigation. Other classes of compounds found in *B. pilosa* are also in need of further examination, as shown in Table 11.

## 3. Pharmacological Properties

As outlined in Table 3, *B. pilosa *is traditionally used to treat a wide variety of ailments. Different preparations of its whole plant and/or parts have been purported to treat over 40 categories of illnesses. Scientific studies, although not extensive, have demonstrated that *B. pilosa *extracts and/or compounds have antitumor [28–36], antiinflammatory [18, 32, 37–42], antidiabetic and antihyperglycemic [7, 43–46], antioxidant [47–49], immunomodulatory [29, 50], antimalarial [30, 51], antibacterial [51–53], antifungal [53, 54], antihypertensive, vasodilatory [19, 55], and antiulcerative [17] activities. In this section, the primary pharmacological properties of *B. pilosa* extracts and phytochemicals are presented and discussed.

### 3.1. Anticancer Activity

Folkloric reports revealed the possible antitumor efficacy of *B. pilosa*, and several scientific *in vitro *studies have supported the claim that *B. pilosa* extracts and isolated compounds possess anti-cancer activities against a variety of cancer cells. Several studies have used bioassay guided isolation and fractionation methods to discover new compounds from *B. pilosa*. For example, Kviecinski and colleagues tested hydroalcoholic crude extracts, chloroform, ethyl acetate, and methanol fractions for anti-tumor activity [35]. The cytotoxicity of the extracts was assessed using brine shrimp, hemolytic, MTT, and neutral red uptake (NRU) assays. *In vivo* studies were performed using Ehrlich ascites carcinoma in isogenic BALB/c mice. Among them, the chloroform fraction was the most toxic with a half maximal inhibitory concentration (IC_50_) of 97 ± 7.2 and 83 ± 5.2 *μ*g/mL in NRU and MTT, respectively [35]. Kumari and colleagues also reported the anti-cancer and anti-malarial activities of *B. pilosa* leaves [30]. Based on a cytotoxicity-directed fractionation strategy, they identified phenyl-1,3,5-heptatriene with IC_50_ values of 8 ± 0.01, 0.49 ± 0.45, 0.7 ± 0.01, and 10 ± 0.01 *μ*g/mL against human oral, liver, colon, and breast cancer cell lines, respectively. However, phenyl-1,3,5-heptatriyne showed lower activity against breast cancer cell lines than the chloroform leaf extract which had an IC_50_ value of 6.5 ± 0.01 *μ*g/mL. Moreover, the positive control, taxol, showed higher activity than phenyl-1,3,5-heptatriyne [30]. Furthermore, *in vitro* comet assays were performed to evaluate the toxicity of n-hexane, chloroform, and methanol extracts of *B. pilosa* and its ethyl acetate, acetone, and water fractions on Hela and KB cells. The ethyl acetate fraction from the methanol extract exhibited the highest activity with half maximal cytotoxic concentrations (CTC_50_) of 965.2 *μ*g/mL and 586.2 *μ*g/mL against Hela and KB cells, respectively. Despite the moderate toxicity, these findings suggest that these *B. pilosa* extracts/fractions could be useful for future studies [36]. Hot water extracts of *B. pilosa* var. *minor* Sheriff were also assessed for its antileukemic effects on leukemic cell lines L1210, U937, K562, Raji, and P3HR1 using XTT-based colorimetric assays. The extract inhibited the five cell lines with IC_50_ values ranging from 145 *μ*g/mL to 586 *μ*g/mL. L1210, K562, Raji, and P3HR1 were more sensitive to *B. pilosa* extract with IC_50_ values below 200 *μ*g/mL [101]. 

Consistent with the antitumor activities of *B. pilosa* extracts and fractions, some of its phytochemicals also showed anticancer activity as outlined in Table 11. Among them, luteolin (**103**), a well-studied flavonoid with multiple bioactivities, was more effective against tumor cell proliferation than its derivatives with IC_50_ values ranging from 3 *μ*M to 50 *μ*M in cells, and 5 to 10 mg/kg in animals. Luteolin was also found to fight cancer as a food additive at concentrations of 50 to 200 ppm [31] and prevent skin cancer [31] and cancer invasion [102]. Lee and colleagues reported that luteolin prevents cancer by inhibiting cell adhesion and invasion [102]. Significant inhibition concentration was reported to be 5 *μ*M and complete inhibition concentration was reported to be 40 *μ*M. Moreover, luteolin was reported to inhibit hepatocyte growth factor (HGF)-induced cell scattering as well as cytoskeleton changes such as filopodia and lamellipodia which was determined using phase-contrast and fluorescence microscopy. Furthermore, luteolin also inhibited the HGF-induced phosphorylation of c-Met, ERK 1/2, and Akt as well as the MAPK/ERK and P13 K-Akt pathways [31, 102]. Other mechanisms underlying the anticancer activities of luteolin are the inhibition of topoisomerase I and II, which inhibits cell replication and DNA repair thus promoting apoptosis, regulation of PI-3-Kinase/Akt/MAPK/ERK/JNK, activation of apoptosis in the mitochondrial pathway by activating caspase 9 and caspase 3, which were found in malignant cells but not in normal human peripheral blood mononuclear cells, death receptor-induced apoptosis, and a cell-cycle arrest mechanism, inhibition of fatty acid synthase which is upregulated in many cancer cells, and sensitization to chemotherapy whereby luteolin increases the susceptibility of cancer cells to chemotherapy [31]. Butein (**82**) is another flavonoid that showed a cytotoxic effect on human colon adenocarcinoma cell proliferation with a reported IC_50_ value of 1.75 *μ*M. Butein at 2 *μ*M affected the incorporation of [^14^C]-labeled leucine, thymidine, and uridine which can cause the inhibition of DNA, RNA, and protein synthesis of human colon cancer cells. Moreover, butein also exhibited noncompetitive inhibition of 1-chloro-2,4-dinitrobenzene (CDNB) in glutathione S-transferase (GST) activity. Tumor resistance was correlated with high levels of GST, thus, butein inhibited proliferation of cancer cells [33]. Another flavonoid present in *B. pilosa*, centaureidin (**109**), also showed anti-cancer activity in B lymphoma cells. Centaureidin, isolated from *Polymnia fruticosa*, inhibited tubulin polymerization *in vitro* and induced mitotic figure formation in CA46 Burkitt lymphoma cells. Using turbidimetric assay, the IC_50_ value of centaureidin in inhibition of mitosis was 3 *μ*M [103]. Cytotoxicity of centaureidin was further analyzed using American National Cancer Institute (NCI) 60 human tumor cell lines. The cytotoxicity potency of centaureidin, expressed as GI_50_ (50% growth inhibition in the NCI tumor line panel), was 0.24 *μ*M [34]. These data mark centaureidin as a promising antimitotic agent for tumor therapy. 

In addition to anti-tumor flavones, polyynes found in *B. pilosa* have also been shown to possess anti-tumor properties. Based on a bioactivity-directed isolation approach, Wu and colleagues identified two polyyne aglycones from the ethyl acetate fraction of *B. pilosa *[70]. 1,2-Dihydroxytrideca-5,7,9,11-tetrayne (**48**) and 1,3-Dihydroxy-6(*E*)-tetradecene-8,10,12-triyne (**46**) exhibited significant anticell proliferation activity in primary human umbilical vein endothelium cells (HUVEC) with IC_50_ values of 12.5 *μ*M and 1.73 *μ*M, respectively. They also decreased angiogenesis and promoted apoptosis in human endothelial cells. Their anti-angiogenic and cytotoxic effects correlated with activation of the CDK inhibitors and caspase-7 [70]. In addition, 1,2-Dihyroxy-5(*E*)-tridecene-7,9,11-triyne (**45**) showed antiangiogenic effects in HUVECs with an IC_50_ value of 12.4 *μ*M as evidenced by a decrease in the tube formation and migration of HUVECs [28]. The IC_50_ value of compound **45** in the inhibition of basic fibroblast growth factor-induced HUVEC growth was 28.2 *μ*M. However, it had higher IC_50_ values than those for lung carcinoma cells and keratinocytes. This compound could also inhibit cell proliferation of HUVECs, lung carcinoma A549 cells and HACAT keratinocytes. The mechanism by which compound **45** inhibits HUVEC growth and angiogenesis is complicated and includes decreasing the expression of cell cycle regulators (CDK4, cyclins D1 and A, retinoblastoma (Rb), and vascular endothelial growth factor receptor 1), caspase-mediated activation of CDK inhibitors p21 (Cip1) and p27 (Kip), upregulation of Fas ligand expression, downregulation of Bcl-2 expression, and activation of caspase-7 and poly (ADP-ribose) polymerase [28]. 

### 3.2. Anti-Inflammatory Activity

*B. pilosa* is commonly used to treat inflammatory disorders. The anti-inflammatory phytochemicals present in *B. pilosa *are listed in Table 11. Cyclooxygenase-2 (COX-2) is a physiologically important enzyme that converts arachidonic acid to prostaglandin (PGE_2_). Its expression is induced by a wide variety of external stimuli indicating its involvement in inflammatory diseases, and it is used as an inflammatory marker [42]. Yoshida and colleagues studied the effects of the aqueous extracts of *B. pilosa* aerial parts in the production of COX-2 and PGE_2_ as well as on the activation of mitogen activated protein kinases (MAPKs) in normal human dermal fibroblasts (HDFs) in response to inflammatory cytokine, IL-1*β*. This work showed that IL-1*β* activated MAPKs such as ERK1/2, p38, and JNK to different extents and induced COX-2 expression. The COX-2 expression in HDFs was regulated mainly by p38 following IL-1*β* stimulation. Consistently, the p38 inhibitor SB203580 blocked this expression. Using this cell platform, *B. pilosa* extracts were tested for inhibition of inflammation. The extract dose-dependently suppressed the activation of p38 and JNK and moderately suppressed ERK1/2, as well as suppressing COX-2 expression and PGE_2_ production [42]. This work supports the use of *B. pilosa* as an anti-inflammatory agent; however, no compounds responsible for the anti-inflammatory activity of *B. pilosa* were identified. 

A further study also reported the anti-inflammatory activity as well as the antiallergic activity of *B. pilosa *[37]. In this study, dried powder of the aerial part of *B. pilosa*, which had been pretreated with the enzyme cellulosine, was used for further tests. The results showed that oral administration of the cellulosine-treated *B. pilosa* lowered the level of serum IgE in mice 10 days after immunization with DNP (2,4-dintrophenyl)-Ascaris as an antigen. This treatment also reduced dye exudation in skin induced by passive cutaneous anaphylaxis and production of inflammatory mediators, histamine, and substance P in rats [37]. Phytochemical analysis showed that cellulosine treatment increased the percentage of caffeic acid and flavonoids. This study suggests that *B. pilosa* and its phenolics have anti-inflammatory functions.

Phenolics and polyynes are major anti-inflammatory phytochemicals present in *B. pilosa* (Table 11). Unsurprisingly, phenolics such as luteolin (**103**) and ethyl caffeate (**161**) that are major constituents of *B. pilosa* have also been reported to possess anti-inflammatory activity. Luteolin was reported to exhibit anti-inflammatory activity in macrophages. Xagorari and colleagues showed that luteolin inhibited the release of inflammatory cytokines, TNF-*α* and interleukin-6, in RAW 264.7 cells following LPS stimulation [41]. It inhibited TNF-*α* production with an IC_50_ value of 1 *μ*M. The underlying anti-inflammatory mechanism of luteolin was reported to be the inactivation of Akt and NF-*κ*B activation [41]. In addition, luteolin was reported to confer anti-inflammatory activity through inhibition of LPS-stimulated iNOS expression in BV-2 microglial cells. It inhibited LPS-activated microglia in a dose-dependent manner with an IC_50_ value of 6.9 *μ*M. Moreover, immunoblot and RT-PCR data proved that luteolin suppressed I*κ*B-*α* degradation and iNOS expression in LPS-activated microglia [39]. Kim and colleagues stated that luteolin may have beneficial effects on inflammatory neural diseases through inhibition of iNOS expression [39]. A related study revealed that luteolin decreased the transcriptional activity of NF-*κ*B RelA via partial inhibition of TNF-mediated NF-*κ*B DNA binding activity. Luteolin also inhibited Akt phosphorylation and induced degradation of a transcription factor, interferon regulatory factor (IRF) [40]. 

Chiang and colleagues showed that ethyl caffeate (**161**) significantly inhibited NO production in mouse macrophages, RAW 264.7 cells [38]. Based on MTT assays, they concluded that this inhibition was not due to the cytotoxicity of ethyl caffeate. The IC_50_ value of ethyl caffeate in the inhibition of NO production was 5.5 *μ*g/mL, slightly lower than curcumin (positive control) which has an IC_50_ value of 6.5 *μ*g/mL. They demonstrated that ethyl caffeate exerted anti-inflammatory activity via the reduced transcription and translation of iNOS (inducible nitric oxide synthase) in RAW 246.7 cells. In addition, this compound also suppressed COX-2 expression in RAW 246.7 cells and MCF-7 cells. The *in vivo* anti-inflammatory effect of ethyl caffeate was verified by testing in TPA-treated mouse skin. Like celecoxib, the positive control, ethyl caffeate significantly abolished COX-2 expression in a dose-dependent manner. Ethyl caffeate at 1 mg/200 *μ*L/site (24 mM) inhibited COX-2 expression at a level comparable to celecoxib at 1 mg/200 *μ*L/site (13 mM). Remarkably, ethyl caffeate at 48 mM (2 mg/200 *μ*L/site) was more effective than celecoxib at 131 mM (10 mg/200 *μ*L/site). Moreover, this compound inhibited the activation of nuclear factor-*κ*B (NF-*κ*B) by LPS via the prevention of NF-*κ*B binding to DNA [38]. In addition, 3 ethyl caffeate analogs (ethyl 3,4-dihydroxyhydrocinnamate, ethyl cinnamate, and catechol) also showed different degrees of NF-*κ*B binding to DNA as listed in Table 12. Ethyl cinnamate lacks a catechol moiety which results in ineffective inhibition of NF-*κ*B binding to DNA [38]. 

Pereira and colleagues assessed the anti-inflammatory and immunomodulatory activities of *B. pilosa* methanol extract as well as one polyyne, 2-*O*-*β*-glucosyltrideca-11(*E*)-en-3,5,7,9-tetrayn-1,2-diol (**54**) in T lymphocytes and a zymosan-induced arthritis mouse model [18]. They first examined the *in vitro *effect of the *B. pilosa* extract and compound **54 **on cell proliferation of human T cells stimulated with 5 *μ*g/mL phytohemagglutinin (PHA) or 100 nM 12-*O*-tetradecanoyl phorbol-13-acetate (TPA) plus 15 *μ*M ionomycin and on cell proliferation of mouse T cells stimulated with 5 *μ*g/mL concanavalin A (Con A). The data demonstrated that both methanol extract and compound **54** suppressed T-cell proliferation in a dose-dependent manner. The estimated IC_50_ values of the *B. pilosa* extract against human T cells stimulated with 5 *μ*g/mL PHA and 100 nM TPA plus 15 *μ*M ionomycin were 12.5 and 25 *μ*g/mL, respectively. In comparison with the methanol extract, compound **54** showed 10-fold more inhibition of human T-cell proliferation with an estimated IC_50_ value of 1.5 *μ*g/mL. Accordingly, the *B. pilosa* extract and compound **54** dose-dependently suppressed mouse T-cell proliferation with estimated IC_50_ values of 30 and 2.5 *μ*g/mL, respectively. Taken together, the data indicate that the *B. pilosa* extract and compound **54** act on human and mouse T cells. To test the *in vivo* effect of the *B. pilosa* extract and compound **54**, a zymosan-induced arthritis mouse model was used. This model was established from B10.A/SgSnJ mice with an injection of zymosan (0.15 mg). The zymosan-injected mice received an intraperitoneal injection of the *B. pilosa* extract (1, 5, or 10 mg) at one dose a day for 5 days. Popliteal lymph node (PLN) weight was monitored to check the development of arthritis. The results revealed that 10 mg of the methanol extract of *B. pilosa* extract could significantly diminish inflammation as evidenced by PLN weight [18]. This work suggests that *B. pilosa* (and compound **54**) can suppress immune response and inflammation.

### 3.3. Antidiabetic Activity

Anti-diabetic agents are primarily developed from plants and other natural resources [7, 43–46].  *B. pilosa* is one of 1,200 plant species that have been investigated for antidiabetic activity [119, 120]. *B. pilosa *is used as an anti-diabetic herb in America, Africa, and Asia [45, 119, 121]. Many studies have indicated that *B. pilosa* could treat type 1 diabetes (T1D) and type 2 diabetes (T2D) in animals. 

Etiologically speaking, T1D is caused by autoimmune-mediated destruction of pancreatic *β* cells, leading to insulin deficiency, hyperglycemia, and complications. Currently, there is no cure for T1D. Polarization of Th cell differentiation controls the development of T1D. Suppression of Th1 cell differentiation and promotion of Th2 cell differentiation ameliorate T1D [122]. One study showed that the butanol fraction of *B. pilosa* inhibited T-cell proliferation, decreased Th1 cells and cytokines, and increased Th2 cells and cytokines, leading to prevention of T1D in nonobese diabetic (NOD) mice [44]. Based on a bioactivity-directed isolation strategy, 3 polyynes, 2-*β*-D-Glucopyranosyloxy-1-hydroxytrideca-5,7,9,11-tetrayne (**53**), also known as cytopiloyne, 3-*β*-D-Glucopyranosyl-1-hydroxy-6(*E*)-tetradecene-8,10,12-triyne (**69**), 2-*β*-D-Glucopyranosyloxy-1-hydroxy-5(*E*)-tridecene-7,9,11-triyne (**50**) were identified from *B. pilosa* [44, 46]. The IC_50_ value of the butanol fraction was 200 *μ*g/mL. This inhibition was reported to be partially attributed to cytotoxicity because the butanol fraction at 180 *μ*g/mL could cause 50% death of Th1 cells. Moreover, this study suggested that the butanol fraction may prevent diabetes in NOD mice *in vivo* via downregulation of Th1 cells or upregulation Th2 cells that have effects which are antagonistic of those of Th1 cells [44]. This was proven by intraperitoneal injection of the butanol fraction at a dose of 3 mg/kg BW, 3 times a week, to NOD mice from 4 to 27 weeks. This dosage resulted in lower incidence of diabetes (33%). At a dose of 10 mg/kg, the butanol fraction of *B. pilosa* totally eliminated (0%) the initiation of the disease. To further support this result, assessment of IgG2a and IgE production was performed in the serum of NOD mice. As *in vivo* results obtained from intracellular cytokine staining, experiments were not very conclusive levels of IgG2a and IgE were measured since Th1 cytokine IFN*γ*; and Th2 cytokine IL-4 favor the production of IgG2a and IgE, respectively. As expected, high levels of IgE and some decline in the levels of IgG2a were observed in the serum. Profiling of the butanol extract revealed five compounds, 3-*β*-D-Glucopyranosyl-1-hydroxy-6(*E*)-tetradecene-8,10,12-triyne (**69**), 2-*β*-D-Glucopyranosyloxy-1-hydroxy-5(*E*)-tridecene-7,9,11-triyne (**50**), 4,5-Di-*O*-caffeoylquinic acid, 3,5-Di-*O*-caffeoylquinic acid, and 3,4-Di-*O*-caffeoylquinic acid. Only the first two compounds showed similar effects on the prevention of diabetes in NOD mice as the *B. pilosa *butanol fraction. Moreover, compound **50** showed greater activity than compound **69** in terms of enhancement (by 34% compared to 8%) of differentiation of Th0 to Th2 at 15 *μ*g/mL (both compounds) and inhibition (by 40% compared to 10%) of differentiation to Th1 at the same concentration [44]. 

Among the three polyynes found in *B. pilosa*, cytopiloyne (**53**) had the most potent anti-T1D activity [46]. To test the *in vivo* effect of cytopiloyne, NOD mice received intraperitoneal or intramuscular injection of cytopiloyne at 25 *μ*g/kg BW, 3 times per week. Twelve-week-old NOD mice started to develop T1D, and 70% of NOD mice aged 23 weeks and over developed T1D. Remarkably, 12- to 30-week-old NOD mice treated with cytopiloyne showed normal levels of blood glucose (<200 mg/dL) and insulin (1-2 ng/mL). Consistent with T1D incidence, cytopiloyne delayed and reduced the invasion of CD4^+^ T cells into the pancreatic islets [46]. 

*In vitro *study showed that cytopiloyne (**53**) inhibited the differentiation of naïve Th (Th0) cells (i.e., CD4^+^ T cells) into Th1 cells and promoted differentiation of Th0 cells into Th2 cells [50]. The *in vitro* data are consistent with the *in vivo* results indicating that cytopiloyne reduced Th1 differentiation and increased Th2 differentiation as shown by intracellular cytokine staining and FACS analysis [46]. In line with the skewing of Th differentiation, the level of serum IFN-*γ* and IgG2c decreased while that of serum IL-4 and serum IgE increased compared to the negative controls (PBS-treated mice). Cytopiloyne also enhanced the expression of GATA-3, a master gene for Th2 cell differentiation, but not the expression of T-bet, a master gene for Th1 cell differentiation, further supporting its role in skewing Th differentiation [46]. 

Also importantly, cytopiloyne partially depleted CD4^+^ rather than CD8^+^ T cells in NOD mice [46]. As shown in Table 13, coculture assays showed that the depletion of CD4^+^ T cells was mediated through the induction of Fas ligand expression on pancreatic islet cells by cytopiloyne, leading to apoptosis of infiltrating CD4^+^ T cells in the pancreas via the Fas and Fas ligand pathways. However, cytopiloyne did not induce the expression of TNF-*α* in pancreatic islet cells and, thus, had no effect on CD8^+^ T cells [46]. 

In addition, Chang and colleagues showed that cytopiloyne dose-dependently inhibited T-cell proliferation stimulated by IL-2 plus Con A or anti-CD3 antibody, using [^3^H] thymidine incorporation assay [46].

Overall, the mechanism of action of cytopiloyne and, probably, its derivatives in T1D includes inhibition of T-cell proliferation, skewing of Th cell differentiation, and partial depletion of Th cells. Due to the anti-diabetic mechanisms of action, it was hypothesized that cytopiloyne protects NOD mice from diabetes by a generalized suppression of adaptive immunity. To evaluate this hypothesis, ovalbumin (Ova) was used as a T-cell dependent antigen to prime NOD mice, which had already received cytopiloyne or PBS vehicle. Ova priming boosted similar anti-Ova titers in cytopiloyne-treated mice and PBS-treated mice, but a difference in immunoglobulin isotype was observed in the two groups. Thus, it was concluded that cytopiloyne is an immunomodulatory compound rather than an immunosuppressive compound [46, 50]. 

T2D is a chronic metabolic disease with serious complications resulting from defects in either insulin secretion, insulin action, or both [123]. A study by Ubillas et al. showed that the aqueous ethanol extract of the aerial part of *B. pilosa* at 1 g/kg body weight (BW) lowered blood glucose in db/db mice, a T2D mouse model [45]. Based on a bioactivity-guided identification, compounds **69** and **50** were identified. Further, the mixture of the compounds (**69 : 50**) in a 2 : 3 ratio significantly decreased blood glucose concentration and reduced food intake on the second day of treatment when administered at doses of 250 mg/kg twice a day to C5BL/Ks-db/db mice. When tested at 500 mg/kg, a more substantial drop in blood glucose level as well as the stronger anorexic effect (food intake reduced from 5.8 g/mouse/day to 2.5 g/mouse/day) was observed [45]. In this study, it was suggested that the blood glucose lowering effect of *B. pilosa* was caused, in part, by the hunger suppressing effect of its polyynes [45]. However, the hunger suppressing effect of the ethanol extract of *B. pilosa *was not found in the studies described below. In another study [43], water extracts of *B. pilosa *(BPWE) were used in diabetic db/db mice, aged 6–8 weeks, with postprandial blood glucose levels of 350 to 400 mg/dL. Like oral anti-diabetic glimepiride, which stimulates insulin release, one single dose of BPWE reduced blood glucose levels from 374 to 144 mg/dL. The antihyperglycemic effect of BPWE was inversely correlated to an increase in serum insulin levels, suggesting that BPWE acts to lower blood glucose via increased insulin production. However, BPWE had different insulin secretion kinetics to glimepiride [43]. One flaw in current anti-diabetics is their decreasing efficacy over time. The authors investigated the long term anti-diabetic effect of BPWE in db/db mice. BPWE reduced blood glucose, increased blood insulin, improved glucose tolerance, and reduced the percentage of glycosylated hemoglobin (HbA1c). Both long-term and one-time experiments strongly support the anti-diabetic action of BPWE [43]. In sharp contrast to glimepiride, BPWE protected against islet atrophy in mouse pancreas. The investigators further evaluated anti-diabetic properties of 3 *B. pilosa* varieties, *B. pilosa* L. var. *radiate *(BPR), *B.pilosa* L. var. *pilosa *(BPP), and *B. pilosa* L. var. *minor* (BPM) in db/db mice [7]. One single oral dose (10, 50 and 250 mg/kg body weight) of BPR, BPP, or BPM crude extracts decreased postprandial blood glucose levels in db/db mice for up to four hours, and the reduction of glucose levels in the blood appeared to be dose-dependent. Comparing the three variants, BPR extract resulted in a higher reduction in blood glucose levels when administered at the same dose as the other two varieties. In terms of serum insulin levels, a dose of 50 mg/kg of each extract was used, and the BPR extract, together with the three polyynes, significantly increased the serum insulin level in db/db mice. Long-term experiments (28-day treatment) were then conducted using diabetic mice with postprandial glucose levels from 370 to 420 mg/dL, and glimepiride was used as positive control. The range of dosages applied was from 10 mg/kg BW to 250 mg/kg BW. Results showed that the positive control as well as the crude extracts of the three varieties lowered the blood glucose levels in db/db mice. However, only BPR extract, containing a higher percentage of cytopiloyne (**53**), reduced blood glucose levels and augmented blood insulin levels more than BPP and BPM. The percentage of glycosylated hemoglobin A1c (HbA1c) was also measured and found to be 7.9% ± 0.5% in mice aged 10–12 weeks, and 6.6% ± 0.2%, 6.1% ± 0.3% and 6.2% ± 0.3% in the blood of age-matched mice following treatment with BPR crude extract (50 mg/kg), glimepiride (1 mg/kg), and compound **53** (0.5 mg/kg), respectively [7]. Among the polyynes found in *B. pilosa*, cytopiloyne was the most effective against T2D. Hence, cytopiloyne was used for further study on anti-diabetic action and mechanism [124]. The data confirmed that cytopiloyne reduced postprandial blood glucose levels, increased blood insulin, improved glucose tolerance, suppressed HbA1c level, and protected pancreatic islets in db/db mice. Nevertheless, cytopiloyne failed to decrease blood glucose in streptoztocin (STZ)-treated mice whose b cells were already destroyed. Additionally, cytopiloyne dose-dependently increased insulin secretion and expression in b cells as well as calcium influx, diacylglycerol, and activation of protein kinase C*α*. Collectively, the mechanistic studies suggest that cytopiloyne treats T2D via regulation of insulin production involving the calcium/DAG/PKC*α* cascade in b cells. 

The studies detailed above point to the conclusion that cytopiloyne and related polyynes (compounds **69** and **49**) are anti-diabetics in animal models. The data uncover a new biological action of polyynes. It should be noted that, like all anti-diabetic drugs, cytopiloyne failed to prevent or cure diabetes completely but reduced diabetic complications [124]. Intriguingly, 34 polyynes have been found in *B. pilosa* so far. It remains to be seen whether all the polyynes present in this plant have anti-diabetic activities.

### 3.4. Antioxidant Activity

Free radicals can damage cellular components via a series of chemical reactions [48] leading to development and progression of cardiovascular disease, cancer, neurodegenerative diseases and ageing [47]. Free radicals, nitric oxide (NO), and superoxide anions can be produced in macrophages to kill microbes. However, an excessive generation of the free radicals under pathological conditions is associated with a wide range of illnesses. Plants are known to be rich in antioxidant phytochemicals. Chiang and colleagues evaluated the free radical scavenging activity of crude extract, fractions, and compounds of *B. pilosa* using 1,1-diphenyl-2-picrylhydrazyl (DPPH) and hypoxanthine/xanthine oxidase assays [47]. Using DPPH and hypoxanthine/xanthine oxidase assays, they found that the *B. pilosa* crude extract and the ethyl acetate, butanol, and water fractions had free radical scavenging activity. Nine compounds, Heptyl-2-*O*-*β*-xylofuranosyl-(1→6)-*β*-glucopyranoside (**199**), 3-*O*-Rabinobioside (**124**), Quercetin 3-*O*-rutinoside (**130**), Chlorogenic acid (**167**), 3,4-Di-*O*-caffeoylquinic acid (**169**), 3,5-Di-*O*-caffeoylquinic acid (**170**), 4,5-Di-*O*-caffeoylquinic acid (**171**), Jacein (**119**), and Centaurein (**110**) had DPPH radical scavenging activity [47]. The IC_50_ values of the *B. pilosa* crude extract/fractions and compounds are summarized in Tables 14 and 15, respectively. 

Measurement of free radical scavenging activities is one way of assessing the antioxidant activities of *B. pilosa* and its fractions and compounds. It is interesting that the ethyl acetate and butanol fractions are more active than the water fraction and *B. pilosa* crude extract [47]. Of the secondary metabolites, only phenolic compounds **124**, **130**, **167**, **169**, and **171 **showed significant DPPH-radical scavenging activities. Further analysis of the structure-activity relationship of the compounds suggested that substitution of the C3 hydroxyl group with glycosides increased the activity approximately 2-fold (for example, in compounds **124** and **130**) relative to quercetin (–OH in its C3) [47]. Further modification of the structures of the active compounds needs to be performed to test the effects of various substituents on activity. The reason why most of the antioxidant compounds contain phenol moieties in their structure could be that the reduction-oxidation (redox) properties of phenols allow them to act as reducing agents, singlet oxygen quenchers, and hydrogen donors.

A complementary study by Muchuweti and colleagues determined phenolic content, antioxidant activity, and the phenolic profile of *B. pilosa* methanol extract [48]. They estimated that the phenolic content of the methanol extract of *B. pilosa* was 1102.8 ± 2.2 mg/g [48]. Vanillin, hydroxybenzaldehyde, caffeic acid, coumaric acid, and ferulic acid were found in this extract. The *B. pilosa* extract also showed DPPH radical scavenging activity. Furthermore, the antioxidant activity of the flavonoids found in *B. pilosa* was correlated with its hepatoprotective effects through their inhibition of NF-*κ*B activation which may lessen the oxidative stress caused by the production of free radicals during liver injury [81]. This activity might also be due to the anti-inflammatory effects of the aqueous extracts of *B. pilosa* aerial parts on the inhibition of COX-2 and PGE_2_ production [42]. 

Essential oils from *B. pilosa* flowers and leaves are also reported to possess antioxidant activity. With the aim of replacing chemically synthesized additives, Deba and colleagues [53] worked on the antioxidant, antibacterial, and antifungal activities of essential oils and water extracts of *B. pilosa*'s leaves and flowers.  Table 16 summarizes the results obtained from DPPH free radical scavenging assay.

It can be inferred from Table 16 that essential oils from the leaves possessed the highest activity. It is reported elsewhere that monoterpenes present in essential oils such that of *B. pilosa* have protective effects and antioxidant properties [53]. Beta-carotene bleaching method was also performed. Leaves essential oils and aqueous extracts of the leaves and flowers showed higher activity than the flower essential oils. This is due to the volatility of the flower essential oils. The activity exhibited by the aqueous extracts was accounted to the presence of phenolic compounds that are reported to donate a hydrogen atom to free radicals such that the propagation of the chain reaction during lipid oxidation is terminated [53]. Overall, essential oils and phenolics present in *B. pilosa* can be thought of as major antioxidant compounds. 

### 3.5. Immunomodulatory Activity

*B. pilosa* is thought to be an immunomodulatory plant and is reported to be effective in the treatment of immune disorders such as allergy [37], arthritis [37], and T1D [46, 50, 73]. 

As pointed out in the discussion of its anti-diabetic activities (Section 3.3), a combination of phytochemicals method and T-cell activation assays was used to study immunomodulatory properties of *B. pilosa*. IFN-*γ* is a key cytokine released by T and NK cells that mediates immune cells and sustains immunity against pathogens. Defects in IFN-*γ* expression, regulation, and activation result in vulnerability to diseases caused by bacteria and viruses [29]. An elegant study, performed by Chang and colleagues using IFN-*γ* promoter-driven luciferase reporter construct in Jurkat T cells, showed that hot water crude extracts of *B. pilosa* increased IFN-*γ* promoter activity two-fold [29]. Out of the subfractions of this extract, the butanol fraction, but not the water or ethyl acetate, fractions, increased IFN-*γ* promoter activity six-fold. Centaurein (**110**) and centaureidin (**109**) were identified from the butanol fraction and were stated to cause a four-fold increase in IFN-*γ* promoter activity with EC_50_ values of 75 *μ*g/mL and 0.9 *μ*g/mL, respectively. The mechanism of action of centaurein was determined using transcription factors such as AP-1, NFAT, and NF*κ*B which are reported to bind to IFN-*γ* promoter and regulate IFN-*γ* transcription. Unlike with the activity of the positive control, PHA, centaurein caused a four-fold increase in NFAT, a 3-fold increase in NF*κ*B and had little, if any, effect on AP-1 enhancer activities (23-fold, three-fold, and ten-fold increases, respectively, were seen with PHA) [29]. The authors concluded that centaurein modulates IFN-*γ* expression by the NFAT and NF*κ*B pathways. The article only determined the mechanism of action of centaurein. Its aglycone centaureidin may act through the same mechanism though this conclusion needs to be further verified. 

*B. pilosa* extract and its compounds are reported to inhibit differentiation of naïve CD4^+^ helper T (Th0) cells into Th1 cells [44]. Using the Th cell differentiation assay as a screening platform, 3 polyynes, 2-*β*-D-glucopyranosyloxy-1-hydroxytrideca-5,7,9,11-tetrayne (**53**), 3-*β*-D-glucopyranosyl-1-hydroxy-6(*E*)-tetradecene-8,10,12-triyne (**69**), and 2-*β*-D-glucopyranosyloxy-1-hydroxy-5(*E*)-tridecene-7,9,11-triyne (**49**) were discovered from *B. pilosa* [44, 46]. The data shows that cytopiloyne and other two polyynes suppressed the differentiation of type 1 helper T (Th1) cells and production of Th1 cytokines and promoted that of type 2 helper T (Th2) cells and production of Th2 cytokines, thus explaining the immunomodulatory and anti-inflammatory effects of *B. pilosa* and its polyynes. 

Chang and colleagues were the first to report the effect of the butanol extract of *B. pilosa* on the autoimmune diabetes and airway inflammation in mice [73]. Imbalance in the levels of Th1 and Th2 and of various cytokines leads to autoimmune diseases. T1D and other autoimmune diseases (rheumatoid arthritis, Crohn's disease, among others) are exacerbated by an increase Th1 levels (specifically, CD4^+^ Th1 cells) while Th2 cells antagonize this effect [56]. Moreover, Th2 cells mediate asthma in ovalbumin-induced hypersensitivity in BALB/c mice [73]. In their work [73], Chang and colleagues showed that 10 mg/kg butanol extracts with 1.5% (w/w) compound **69** and 1.1% (w/w) compound **49 **(Table 11) ameliorated the development of Th1-mediated diabetes in NOD mice through inhibition of *β* cell death and leukocyte infiltration. At the same dosage, the butanol extracts also exacerbated ovalbumin-induced pulmonary inflammation in BALB/c mice with an increase in the infiltration of eosinophils and mast cells into the airway of the mice [73]. Despite the different outcomes, both mouse models proved the concept that control over the Th1/Th2 shift is associated with autoimmune diseases and that the *B. pilosa* butanol extract can shift the differentiation of Th0 cells to Th2 cells [44, 73]. 

An extended study presented by Chiang and colleagues showed that compound **53** modulates T-cell functions [50]. Using CD4^+^ T cells from BALB/c mice, they demonstrated that cytopiloyne decreased levels of IFN-*γ* producing cells (Th1) by 12.2% (from 72% to 59.8%). Since Th1 and Th2 cell differentiation is antagonistic, it was expected that compound **53** increased the percentage of mouse IL-4 producing cells (Th2) by 7.2% (from 23.7% to 30.9%). Subsequent assessment of effect of compound **53** on the modulation of the transcription of IL-4 and IFN-*γ* showed that cytopiloyne, as expected, decreased the splenocyte levels of IFN-*γ* mRNA and increasing that of IL-4 in a dose-dependent manner. In the range of 0.1 to 3 *μ*g/mL of compound **53**, the effects were not attributed to its cytotoxicity. Consequently, using 3 *μ*g/mL cytopiloyne for 72 and 96 hours, the protein concentration of IFN-*γ* decreased to 18.6% and 44.4%, respectively. Under the same conditions, cytopiloyne increased IL-4 concentrations to 198.5% and 247.0% (for 72 and 96 hours, resp.). This modulation of T-cell differentiation exhibited by compound **53** was used to explain its anti-diabetic activity [50]. 

The anti-diabetic role of cytopiloyne was extensively discussed above (Section 3.3, anti-diabetic activity). The molecular basis of the regulation of cytokine expression by cytopiloyne has been described. Cytopiloyne directly elevated the expression level of IL-4 via GATA-3 upregulation in T cells [46]. However, reduction of IFN-*γ* expression in T cells seemed to come from the indirect opposing effect IL-4 cytokine because the expression level of T-bet was unaltered [46]. In this way, cytopiloyne skewed Th1 polarization into the Th2 state, conferring protection against T1D in NOD mice. Aside from polarization of Th cell differentiation, cytopiloyne also activated the expression of Fas ligand in pancreatic b cells, this increase leading to the partial depletion of T cells and reduction of immune response in local areas such as the pancreas. Of note, cytopiloyne also inhibited T-cell proliferation and activation. By targeting T cells from three immunomodulatory actions, cytopiloyne protects against T1D and probably other Th1-mediated autoimmune diseases [56]. 

The phytochemical constituents of *B. pilosa* exert their functions on different immune cells to modulate immune response. It is possible that some of the compounds may have agonistic or antagonistic effects on immune response. Immune function of *B. pilosa* may depend on its composition and amount of compounds, which could explain the apparently conflicting report of *B. pilosa* butanol extract aggravating allergy in mice [73] while cellulosine-treated extract ameliorated allergy [37, 73]. IFN-*γ* promoter reporter assays and T-cell differentiation assays were used to isolate 2 flavonoids [29] and 3 polyynes [44, 50] as immunomodulatory compounds from the butanol fraction of *B. pilosa*. Interestingly, the flavonoids promote IFN-*γ* expression in NK and T cells. In marked contrast, the polyynes promote IL-4 expression and indirectly inhibit IFN-*γ* expression in differentiating T cells. Sensitivity appears to be the key to identifying structure- and bioactivity-related phytochemicals from medicinal plants.

### 3.6. Antimalarial Activity

The use of chemical drugs against pathogens has resulted in drug-resistant mutants. Examples of drug resistance can be found in the species of the *Plasmodium* that cause malaria. It is important to search for new compounds to combat *Plasmodium* parasites [51]. A study of the anti-malarial activity of the leaf extracts of *B. pilosa* using a combination of phytochemistry and bioassays showed that compound **49 **(Table 11) showed activity against a malaria parasite (*P. falciparum* NF54 strain) with an IC_50_ value of 6.0 *μ*g/mL [30]. In addition, compound **49**, isolated from the aerial parts of *B. pilosa*, inhibited growth of the *P. falciparum* FCR-3 strain with an IC_50_ value of 0.35 *μ*g/mL. This compound was tested for its *in vivo* effect in mice infected with *P. berghei* NK-65 strain. Results showed that compound **49** decreased the average parasitemia in the red blood cells by 20.7 (from 32.8% of that of the control to 12.1%) after an intravenous injection of 0.8 mg/kg BW/day for four days [51]. Further studies addressing the anti-malarial mechanism underlying both polyynes and clinical studies are needed. 

### 3.7. Antibacterial Activity

Emergence of multiple antibiotic-resistant microbes is becoming a global threat to public health and a challenge to disease treatment. For instance, penicillin is commonly used to combat a food-borne intracellular bacterium* Listeria*; however, penicillin-resistant bacteria have been discovered recently [29]. Chang and coworkers isolated centaurein (**110**) and centaureidin (**109**) from *B. pilosa *extract [29, 47]. Centaurein enhances expression of IFN-*γ*, a key cytokine for macrophage activation and, consequently, enhances bactericidal activity in macrophages [29, 47]. In agreement with observed *in vitro* effects, centaurein was reported to prevent and treat *Listeria* infection in C57BL/6J mice [52]. Mechanistic studies confirmed that centaurein exerted antilisterial action via IFN-*γ* expression in wild-type mice but not IFN-*γ* knockout mice [52]. Further *in vitro* studies on centaurein showed that this compound increased IFN-*γ* expression by 13% (from 17% to 20%), 20% (from 21% to 41%), and 11% (from 6% to 17%) in CD4^+^ T cells, CD8^+^T cells, and NK cells, respectively. That is to say, there was an increase in IFN-*γ* producing immune cells. As expected, centaurein also enhanced the expression level of T-bet, a key nuclear factor for IFN-*γ* expression. Consistently, centaurein augmented the serum IFN-*γ* levels in C57BL/6J mice, and this augmentation peaked 24 hours after compound injection. The quantity of mouse serum IFN-*γ* was sufficient to activate macrophages *in vitro* and eradicated GFP-producing *Listeria* inside macrophages. However, the entry of *Listeria *into macrophages was not affected by centaurein-treated mouse sera. Centaurein treatment at 20 *μ*g per mouse rescued 30% of the mice infected with a lethal dose of *Listeria *(2 × 10^6^ CFU). It is noteworthy that in the presence of ampicillin (5 *μ*g/mouse), centaurein (20 *μ*g/mouse) rescued 70% of the mice, suggesting an additive effect between ampicillin and centaurein [52]. Despite lower abundance, centaureidin was 30 times more active than centaurein in terms of IFN-*γ* production [52].

Aside from the indirect antibacterial action mentioned above, extract and/or compounds of *B. pilosa* also showed direct bacteriostatic and/or bactericidal action. One study reported that essential oils and leaf/flower extracts of *B. pilosa* could suppress the growth of gram positive and gram negative bacteria as evidenced by zone of inhibition assays. In this study, antibacterial activity of the essential oils from the leaves and flowers of *B. pilosa* was determined in an attempt to identify natural products as food preservatives for prevention of microbial multiplication and food oxidation. The essential oils and extracts of *B. pilosa* leaves and flowers showed moderate but different extents of antibacterial activity (Table 17). In general, essential oils had higher antibacterial activity than crude extracts. One explanation for this could be that monoterpenes in the essential oils destroy cellular integrity and, subsequently, inhibit the respiration and ion transport processes. The presence of antibacterial *β*-caryophyllene could be another explanation as reported elsewhere [53]. Another study reported that the methanol and acetone extract of *B. pilosa* roots displayed antibacterial activities against the bacteria listed in Table 18 [54] and methanol extracts from the roots seemed to be the most effective. 

Another study indicated that the polyyne, (*R*)-1,2-dihydroxytrideca-3,5,7,9,11-pentayne (**49**), from this plant also suppressed bacterial growth as shown by the minimum inhibitory concentration required to inhibit 50% bacterial growth (MIC_50_) in Table 19. This compound was highly effective against several Gram positive and Gram negative bacteria including the drug-resistant bacteria *Staphylococcus aureus *N315 (MRSA) and *Enterococcus faecalis *NCTC12201 (VRE) [51]. Strikingly, compound **49** had a similar MIC_50_ value to antibiotics (ampicillin, tetracycline, norfloxacin, and amphotericin B) in most of the bacteria tested. 

Antibacterial activity of *B. pilosa* extracts and components, expressed as MIC_50_ and the mean zone of inhibition, is tabulated in Tables 17, 18, and 19. The zone of inhibition for Ampicilline (positive control) ranges from 15.3 ± 0.3 mm to 44.3 ± 0.2 mm [53]. 

### 3.8. Antifungal Activity

*B. pilosa* has traditionally been used to treat microbial infection. Recently, different parts of *B. pilosa* have been tested for antifungal activities. Deba and colleagues first evaluated the antifungal effect of the hot water extracts of the *B. pilosa* roots, stems, and leaves against *Corticium rolfsii*, *Fusarium solani*, and *Fusarium oxysporum*. They discovered that *C. rolfsii* was most suppressed by treatment with *B. pilosa* as its growth was reduced at almost all the tested doses, followed by *F. oxysporum* and *F. solani *[97]. However, the fungicidal activities of the stems, and roots were greater than the leaves [97]. Moreover, the same group assessed the antifungal activity of the essential oils and aqueous extracts from *B. pilosa* flowers and leaves [53]. They showed that the extracts and oils had antifungal activity against *C. rolfsii*, *F. solani*, and *F. oxysporum*. Essential oils appeared to have better fungicidal activity than water extracts as summarized in Table 20. 

Another study by Ashafa and colleagues showed that acetone, methanol, and water extracts of the *B. pilosa* roots showed antifungal activities against *Aspergillus niger*, *A. flavus*, and *Penicillium notatum* using the agar dilution method. The results are tabulated in Table 21 [54]. Negative controls showed 0% growth inhibition. The methanol extract of the *B. pilosa* roots at 10 mg/mL was also effective against* Candida albicans *[54]. Of note, *B. pilosa* obtained from Papua New Guinea had no activity against *A. niger* and *C. albicans *[125], but the South African (Eastern Cape) ecotype exhibited moderate activity against *C. albicans *[54]. This discrepancy may depend on extraction solvents, extraction procedure, assay techniques, different plant parts, and abundance of active compounds.

*B. pilosa* produces a variety of secondary metabolites such as flavonoids, phenylacetylenes, alkaloids, sterols, terpenoids, and tannis [53, 54]. However, none of them have been confirmed as active compounds against fungi. Further investigation of active compounds from *B. pilosa* is necessary to further understand the antifungal efficacy of this plant.

### 3.9. Hypotensive and Vasodilatory Activities

In early studies, Dimo and colleagues used three rat models, normotensive Wistar rats (NTR), salt-loading hypertensive rats (SLHR), and spontaneous hypertensive rats (SHR) to investigate the hypotensive effect of the methanol crude extract of *B. pilosa* leaves [19, 126]. The extract lowered systolic blood pressure in hypertensive rats (SLHR and SHR) to a greater degree than NTR [19, 126]. In addition, a decrease in urinary sodium ions and an increase in urinary potassium ions were observed after treatment with the methanol extract of *B. pilosa* leaves although neither differences were statistically significant [19, 126]. Taking the data together, the study proposed that *B. pilosa* leaf extract reduced blood pressure via vasodilation [19, 126]. The same group continued to test the antihypertensive effect of aqueous and methylene chloride extracts of *B. pilosa* leaves in a hypertensive rat model [19, 126]. To establish a fructose-induced hypertension model, male Wistar rats were given 10% fructose solution to drink *ad libitum* for three weeks. In addition to free access to 10% fructose, the rats were treated with the aqueous (150 mg/kg) or methyl chloride (350 mg/kg) extracts of *B. pilosa* for additional three weeks [19, 126]. Both extracts of *B. pilosa* leaves had a hypotensive effect on rats. However, neither extracts reversed the elevation of serum insulin in fructose-fed rats. Therefore, *B. pilosa* lowered blood pressure irrespective of insulin [19, 126]. To better understand the hypotensive mechanism, the authors investigated the effect of a neutral extract of *B. pilosa* (NBP), a mixture of methanol and methylene chloride (1 : 1) extract after neutralization with NaOH and HCl, on the heart and the blood pressure of NTR and SHR [109]. This study showed that an intravenous injection of the NBP resulted in a biphasic reduction in systolic blood pressure. In addition, one intravenous dose of the extract at 10, 20, and 30 mg/kg BW decreased systolic blood pressure in normal rats by 18.3%, 42.5%, and 30%, respectively, and the same doses reduced the blood pressure in hypertensive rats by 25.8%, 38.9%, and 28.6%, respectively. Only the highest dose (30 mg/kg) affected the force of the contraction of the heart. Atropine and propranolol were used to interfere with the hypotensive action of the NBP. Atropine reduced the initial phase of the hypotensive response in NBP and completely abolished the second phase of hypotensive response in NBP. In contrast, propranolol increased the first hypotensive response but partially abolished the second hypotensive response provoked by the NBP [109]. This mechanistic study suggested that *B. pilosa* invokes the biphasic hypotensive responses via targeting cardiac pump efficiency during the first phase and vasodilation at the second phase [109].

A further study was performed to investigate the relaxing effect of a neutral extract of *B. pilosa* (NBP) on rat aorta contracted with KCl (60 mM) and norepinephrine (0.1 mM) [55]. Cumulative addition of NBP relaxed the rat aorta previously contracted by KCl in a dose-dependent manner. The EC_50_ value of the NBP for vasorelaxation was 0.32 mg/mL. The data also showed that the NBP reduced the contraction of aorta previously contracted by KCl irrespective of the presence of aortic endothelium [55]. 

Pretreatment with glibenclamide, an ATP-dependent K^+^ channel blocker, did not considerably affect the relaxant effect of the NBP on KCl-induced contraction, suggesting that the vasodilatory effect of *B. pilosa* was not related to the opening of this ATP-dependent K^+^ channel [55]. On the other hand, in the presence of indomethacin or pyrilamine maleate, the relaxant response induced by the plant extract was significantly inhibited at the lower concentrations. The plant extract was able to reduce the aorta resting tone, inhibit the KCl-induced contractions by 90% at 1.5 mg/mL and the CaCl_2_-induced contractions by 95% at 0.75 mg/mL. These results demonstrate that *B. pilosa* can act as a vasodilator probably via acting as a calcium antagonist [55]. 

However, no specific compound for the above activity has been identified from *B. pilosa* to date. A bioactivity-guided identification approach may be adopted to identify the active compounds in *B. pilosa* that possess hypotensive and vasodilatory effects and understand their mechanism of action.

### 3.10. Wound Healing Activity

*B. pilosa* has been traditionally used to treat tissue injury in Cameroon, Brazil, and Venezuela [17]. Hassan and colleagues investigated the wound healing potential of *B. pilosa* in Wistar rats [127]. Mirroring the positive control neomycin sulfate, the ethanol extract of *B. pilosa* had faster wound closure than control rats 3, 6, and 9 days after topical application. Histological examination also revealed better collagenation, angiogenesis, and organization of wound tissue seven days after application. Epithelialization and total healing time in *B. pilosa*-treated rats were comparable to those of neomycin sulfate. Together, these data suggest that *B. pilosa* may be a viable alternative to neomycin lotion for the treatment of wounds.

In addition to studying the wound healing effect of *B. pilosa* on external ulcers, Tan and colleagues also examined the effect of methanol, cyclohexane, and methyl chloride extracts of *B. pilosa* on gastric ulcers in Wistar rats fed with 1 mL HCl/ethanol gastric necrotizing solution (150 mM HCl in 60% ethanol), and macroscopically visible lesions were scored [17]. Among the three extracts, methylene chloride extracts exhibited the highest activity showing 46.4% inhibition of lesion formation at a dose of 500 mg/kg BW and complete inhibition at 750 mg/kg [17]. The efficacy of the ethylene chloride extract was followed by that of the methanol extracts which had inhibition ranging from 30.4% to 82.2% at concentrations of 500 mg/kg and 1000 mg/kg BW, respectively [17]. The cyclohexane extracts showed the lowest activity against gastric ulcers in rats with 13.3%, 40%, and 79.7% inhibition at 500, 750, and 1000 mg/kg BW, respectively [17]. To better understand the mode of action of the methylene chloride extract of *B. pilosa*, rats were pretreated with indomethacin, a COX-2 inhibitor involved in prostaglandin synthesis. Pretreatment significantly reduced the protection against HCl/ethanol-induced ulcers to 31.3% inhibition at 750 mg/kg BW, suggesting a link between the antiulcerative activity of *B. pilosa* and prostaglandin synthesis. Unexpectedly, the methylene chloride extract of *B. pilosa* showed little gastric mucosal protection against gastric lesions induced by 95% ethanol (1 mL) [17]. Absolute alcohol is known to cause mucosal/submucosal tissue destruction via cellular necrosis and the release of tissue-derived mediators (histamine and leukotriene C4). Thus, these data imply that *B. pilosa* did not prevent the generation or the necrotic action of these mediators on the gastric microvasculature. In addition, pylorus ligation can increase gastric acid secretion without an alteration of mucosal histamine content. The methylene chloride extract of *B. pilosa* did not possess antisecretory activity. On the contrary, it was observed that increases in the dose of the extract led to elevated gastric juice acidity [17]. Results with both absolute ethanol and pylorus ligation rat models suggested the possibility that the ineffectiveness of *B. pilosa* against gastric ulcers was due to lack of antihistaminic activity in the plant. In summary, overall the data suggest that *B. pilosa* protects against HCL/ethanol-mediated ulcers via inhibition of prostaglandin biosynthesis. 

Previous phytochemical studies showed that a group of flavonoids, acyclichalcones, are present in *B. pilosa *[128, 129] and the chalcones were proposed to have anti-ulcerative activity [130]. Moreover, nine hydroxychalcones were reported to possess gastric cytoprotective effects with 2,4-dihydroxychalcone being the most active [131]. Since methylene chloride extracts appear to be the most active *B. pilosa* extracts, next, the specific anti-ulcerative phytochemicals in the methylene chloride extracts of *B. pilosa* and their modes of action needs to be probed.

Despite the claims listed in Table 3, relatively few scientific studies have been conducted *in vitro* and *in vivo* to address the traditional ethnomedical uses of *B. pilosa*. Information about the use of *B. pilosa* as a botanical therapy recorded so far is far from complete. Studies conducted thus far only serve as a starting point for further investigation of *B. pilosa*, and the ultimate efficacious use of the herb in clinical applications. 

## 4. Toxicology

Despite its use as an ingredient in food for human consumption, studies on systemic toxicity (e.g., acute, subacute, chronic and subchronic toxicities) of* B. pilosa* in humans and animals are still inadequate and insufficient. So far, acute, and/or subchronic toxicities have been evaluated in rats and mice. Oral acute and 28-day toxicities of water extract of *B. pilosa* leaves were evaluated in Wistar rats [132]. An oral dose of water extract of *B. pilosa* leaves at 10 g/kg BW showed no obvious mortality or changes in the appearance in rats [133]. The same extract at 0.8 g/kg BW/day, once a day, showed no obvious sub-chronic toxicity in rats over 28 days, as measured by survival rate, body weight, and gross examination of organs [133]. These data are consistent with our data indicating that oral delivery of the water extract of the *B. pilosa *whole plant at 1 g/kg BW/day, once a day, is safe in rats over 28 days (unpublished data). Taken together, these studies suggest that ingestion of *B. pilosa *aqueous extract at up to at 1 g/kg BW/day, once a day, is highly safe in rats. In addition, the acute toxicity of aqueous and ethanol extracts of *B. pilosa* in mice have been reported [133]. Five- to six-week-old mice with weights between 28 and 35 g received a peritoneal injection of both extracts at the different doses. The LD_50_, the dose that causes 50% lethality, of the aqueous and ethanol extracts in mice was 12.30 g/kg BW and 6.15 g/kg BW, respectively [133]. A complete toxicological study has not been completed for humans. Furthermore, the drug interactions of *B. pilosa* with other drugs are unknown. Further safety verification and clinical trials should be performed before *B. pilosa* can be considered for medicinal use.

## 5. Conclusions

*B. pilosa* is an erect, perennial plant with green leaves, white or yellow flowers and tiny black seeds. As it is distributed worldwide and is widely used as a folk remedy, *B. pilosa* can be thought of as an extraordinary source of food and medicine. However, a comprehensive up-to-date review of research on *B. pilosa* has hitherto been unavailable. In this article, scientific studies on *B. pilosa* have been summarized and critically discussed from the perspectives of botany, ethnomedicine, phytochemistry, pharmacology, and toxicology. *B. pilosa* is claimed to treat more than 40 disorders, and 201 compounds have been identified from this plant. The medicinal utility of *B. pilosa* and its modes of action in relation to its known phytochemicals were discussed herein. Polyynes, flavonoids, phenylpropanoids, fatty acids, and phenolics are the primary bioactive compounds of *B. pilosa*, and they have been reported to be effective in the treatment of tumors, inflammation/immune modulation, diabetes, viruses, microbes, protozoans, gastrointestinal diseases, hypertension, and cardiovascular diseases. Caution should be exercised in the therapeutic use of *B. pilosa* for hypoglycemia, hypotension, bleeding, and allergy.

## Figures and Tables

**Table 1:** Taxonomy of *B. pilosa* [6].

Kingdom	Plantae
Subkingdom	Tracheobionta
Superdivision	Spermatophyta
Division	Magnoliophyta
Class	Magnoliopsida
Subclass	Asteridae
Order	Asterales
Family	Asteraceae
Genus	*Bidens *
Species	*Bidens Pilosa L. *

**Table 2:** Nutritional facts about *B.  pilosa*, courtesy of the United Nations Food and Agriculture Organization [15].

Plant (100 g)	Energy (kcal)	Moisture (%)	Protein (g)	Fat (g)	Carbohydrate (g)	Fiber (g)	Ash (g)	Calcium (mg)	Phosphorus (mg)	Iron (*µ*g)	Carotene equivalent (*µ*g)	Thiamine (mg)
Raw	43	85.1	3.8	0.5	8.4	3.9	2.2	340	67	—	1 800	—
Dried	33	88.6	2.8	0.6	6	1.3	2	111	39	2.3	—	—

“—” denotes not detectable.

**Table 3:** Ethnomedical information about *B. pilosa*.

Disorder	Plant part^b^	Mode of use	Region/Country	References
Stomachache	LE	Decoction	India Africa	[23, 25, 56]
Colics	WP	Decoction	Africa China	[25]
Catarrh	WP	Juice or decoction; taken orally	Cuba	[24]
Diarrhea	LE and WP	Fresh leaves or Decoction	Africa Uganda	[25, 57]
Constipation	WP	Decoction	Africa	[25]
Dysentery/Bacillary dysentery	WP	Decoction	Africa China	[25]
Choleretic	WP	Decoction	Middle America	[25]
Anti-inflammatory	WP	Not stated	China Cuba	[22, 24]
Asthma	WP	Decoction or maceration; taken orally	Cuba China	[21, 24]
Antirheumatic	RT and WP	Juice and decoction	Hong Kong Zulu, Africa	[22, 25]
Acute appendicitis	WP	Decoction	Hong Kong	[21]
Enteritis	WP	Decoction	Africa China	[25]
Pruritus	WP	Decoction	Hong Kong	[21]
Conjunctivitis	WP	Decoction	Africa China	[25]
Otitis	WP	Decoction	Africa China	[25]
Pharyngitis	WP	Decoction	Africa China	[25]
Gastritis	WP	Juice; taken orally	Cuba	[24]
Diabetes	WP	Decoction; taken orally	Cuba Taiwan	[24, 43]
Headache	WP	Decoction	Bafia, Cameroon	[26]
Diuretic	WP	Decoction	Middle America	[25]
Hypotensive	WP	Decoction; taken orally	Bafia, Cameroon	[26]
Colds	LE and WP	Fresh or decoction	China Middle America Uganda	[21, 25, 57]
Yellow Fever	LE and WP	Fresh or decoction	China Middle America Uganda	[21, 22, 25, 57]
Influenza	LE and WP	Fresh or decoction	China Middle America Uganda	[21, 25, 57]
Acute infectious hepatitis	WP	Decoction	Hong Kong	[21]
Intestinal worms	WP	Decoction	Africa	[25]
Malaria	RT and WP	Juice	Africa China	[21, 25]
Eye Infection	LE and WP	Fresh or juice	Uganda Middle America	[25, 57]
Antimicrobial	AP	Decoction for drinking; bathing/external use	Trinidad and Tobago	[22]
Pulmonary tuberculosis	WP	Decoction or maceration; taken orally	Cuba China	[21, 24]
Bacterial infections in gastrointestinal tracts	WP	Decoction	Trinidad and Tobago	[22]
Renal infection	LE	Decoction; taken orally	Cuba	[24]
Sore throat	LE and WP	Fresh or decoction	China Middle America Uganda	[21, 25, 57]
Cough	WP	Decoction; taken orally	Cuba China	[21, 24]
Coolness of the uterus	WP	Decoction; taken orally	Cuba	[24]
Menstrual irregularities	WP	Decoction; taken orally	Cuba	[24]
Dysmenorrhea	WP	Decoction	Bafia, Cameroon	[26]
*Hyperemesis gravidarum* (morning sickness)	WP	Decoction	Africa	[25]
Hemorrhoids	WP	Decoction	Hong Kong	[21]
Nose bleeds	LE and WP	Fresh or decoction	China Middle America Uganda	[21, 25, 57]
Stomach ulcers	LE and WP	Maceration or juice; taken orally	Cuba Middle America Uganda	[21, 24, 25, 57]
Cuts, burns, and skin problems	LE and WP	Fresh plant or decoction; topical application/bathing	Trinidad and Tobago Africa China Cameroon Brazil Venezuela	[17, 22, 25]
Wounds	WP	Crushed herb	China Africa Central America Hawaii	[21]
Snake bites	WP	Pulverized herb	China	[21]

^b^LE: leaves, ST: stem, FW: flower, WP: whole plant, RT: roots, AP: aerial parts.

**Table 4:** Aliphatic natural products isolated from *B.  pilosa* [27].

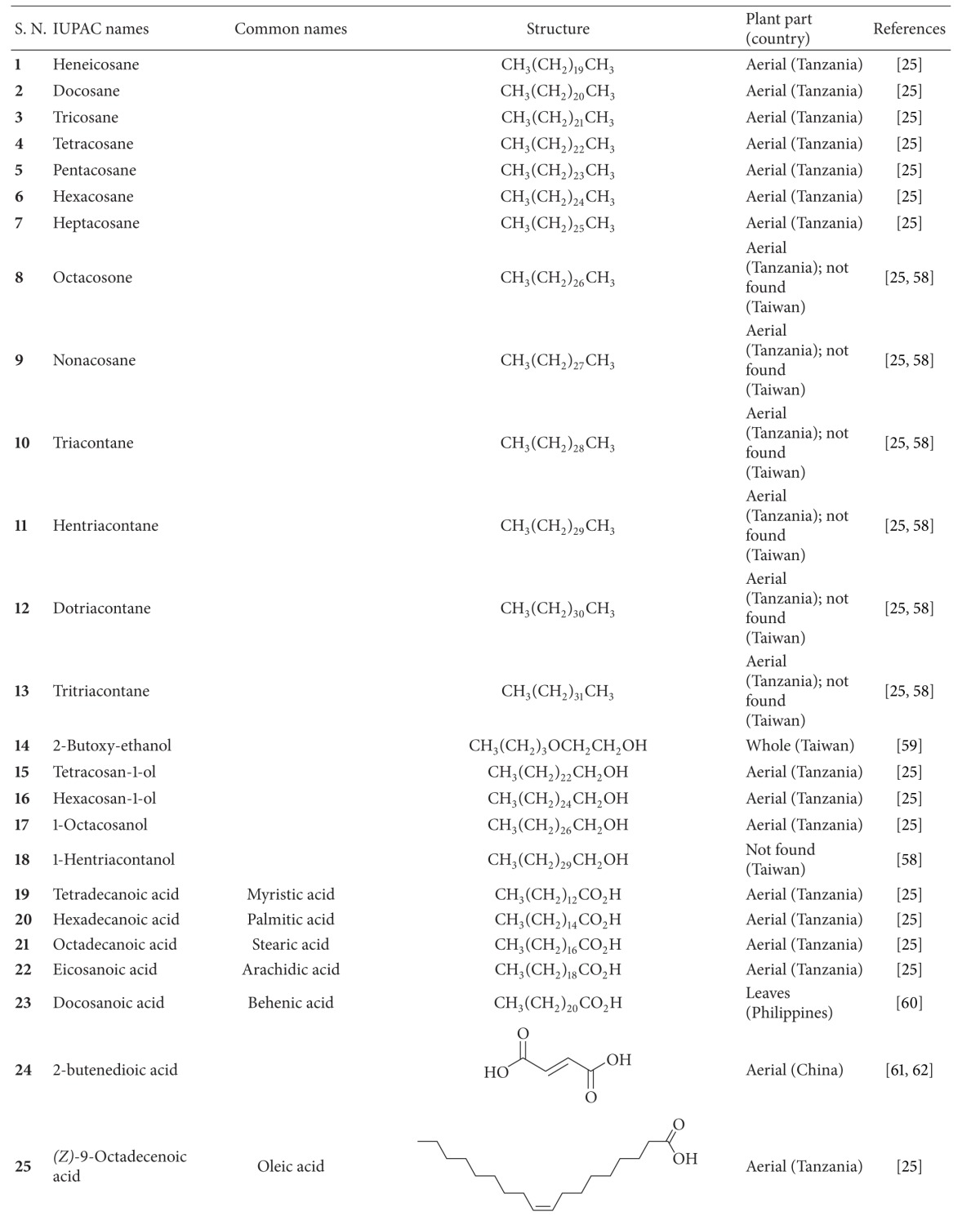 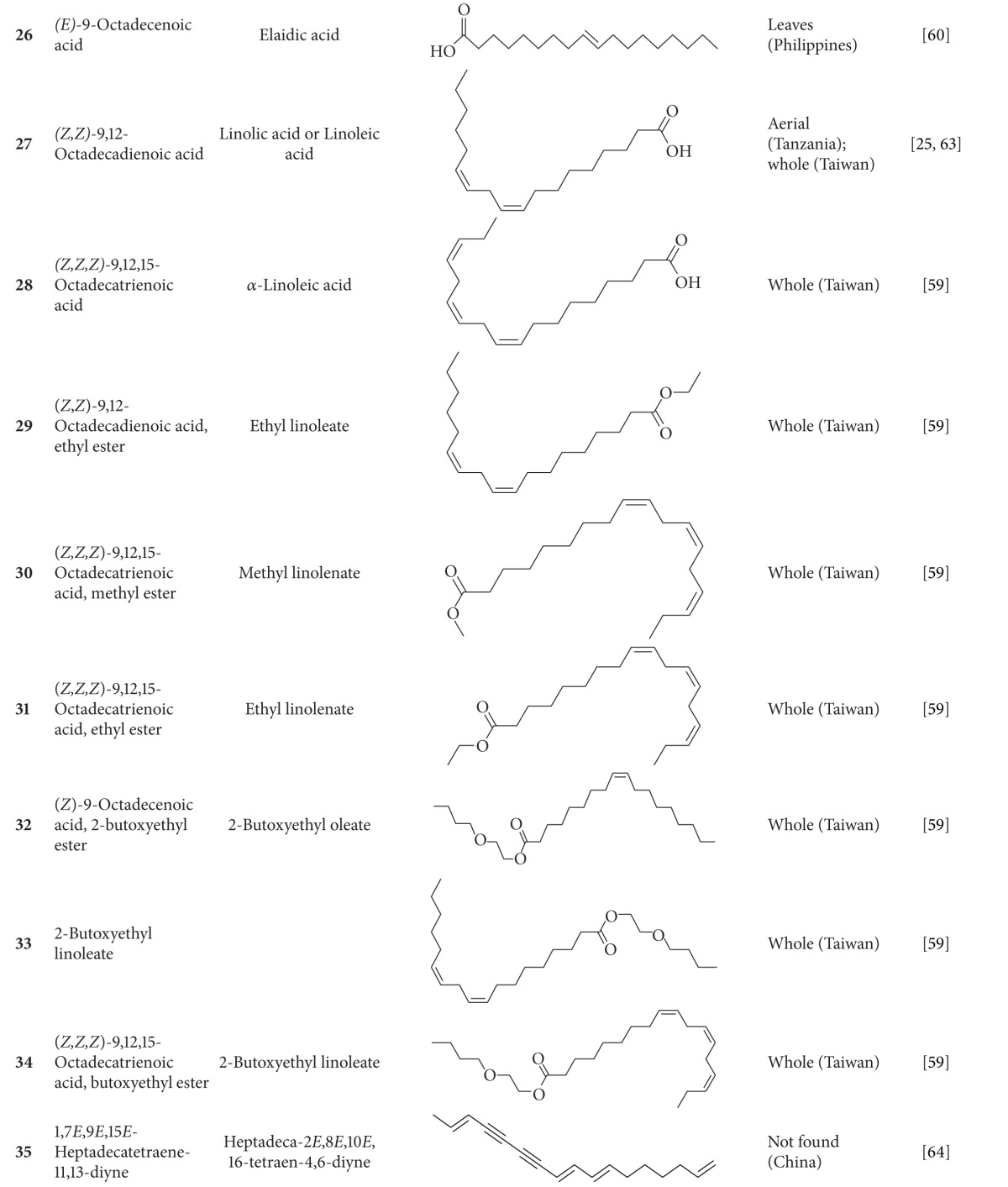 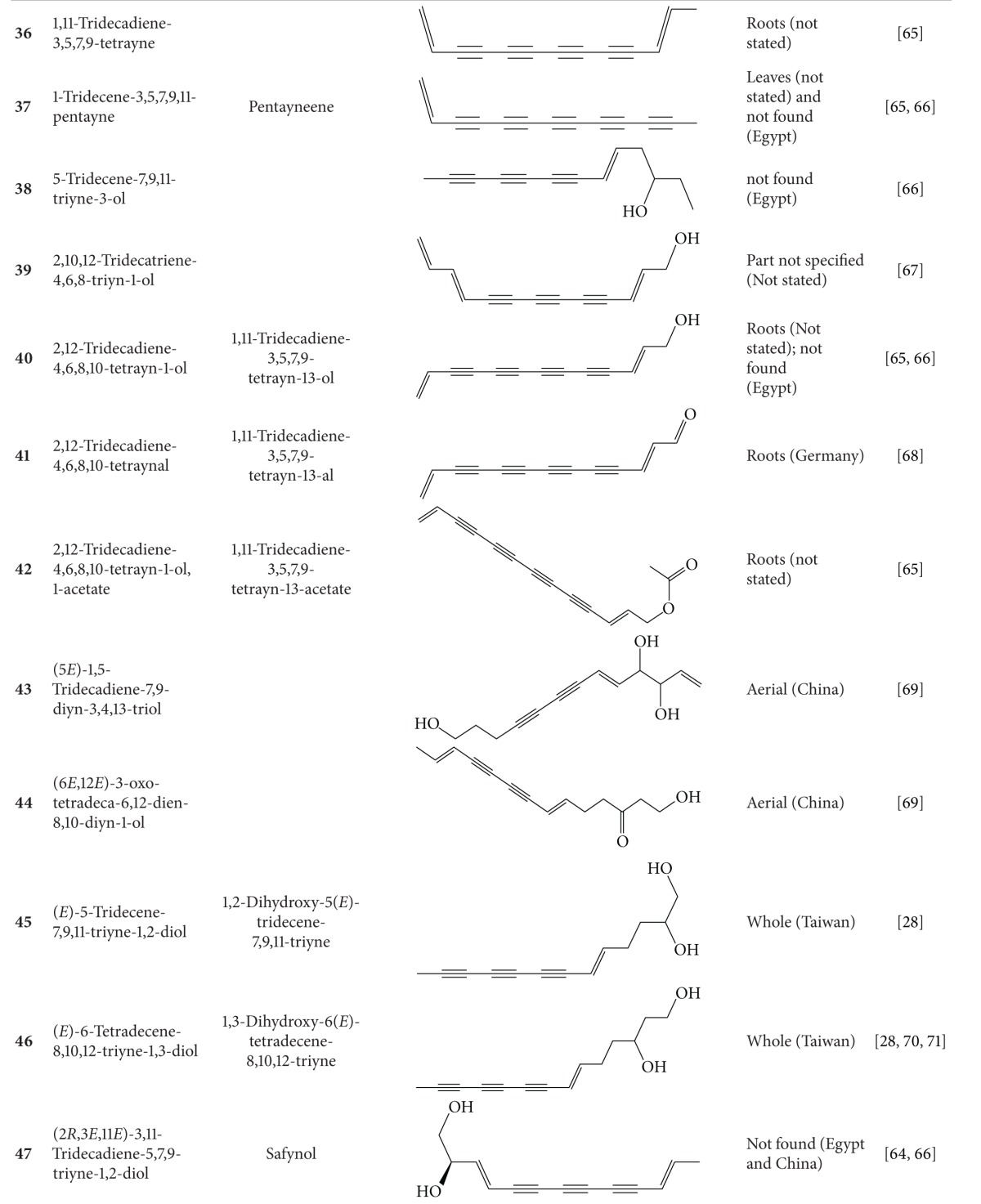 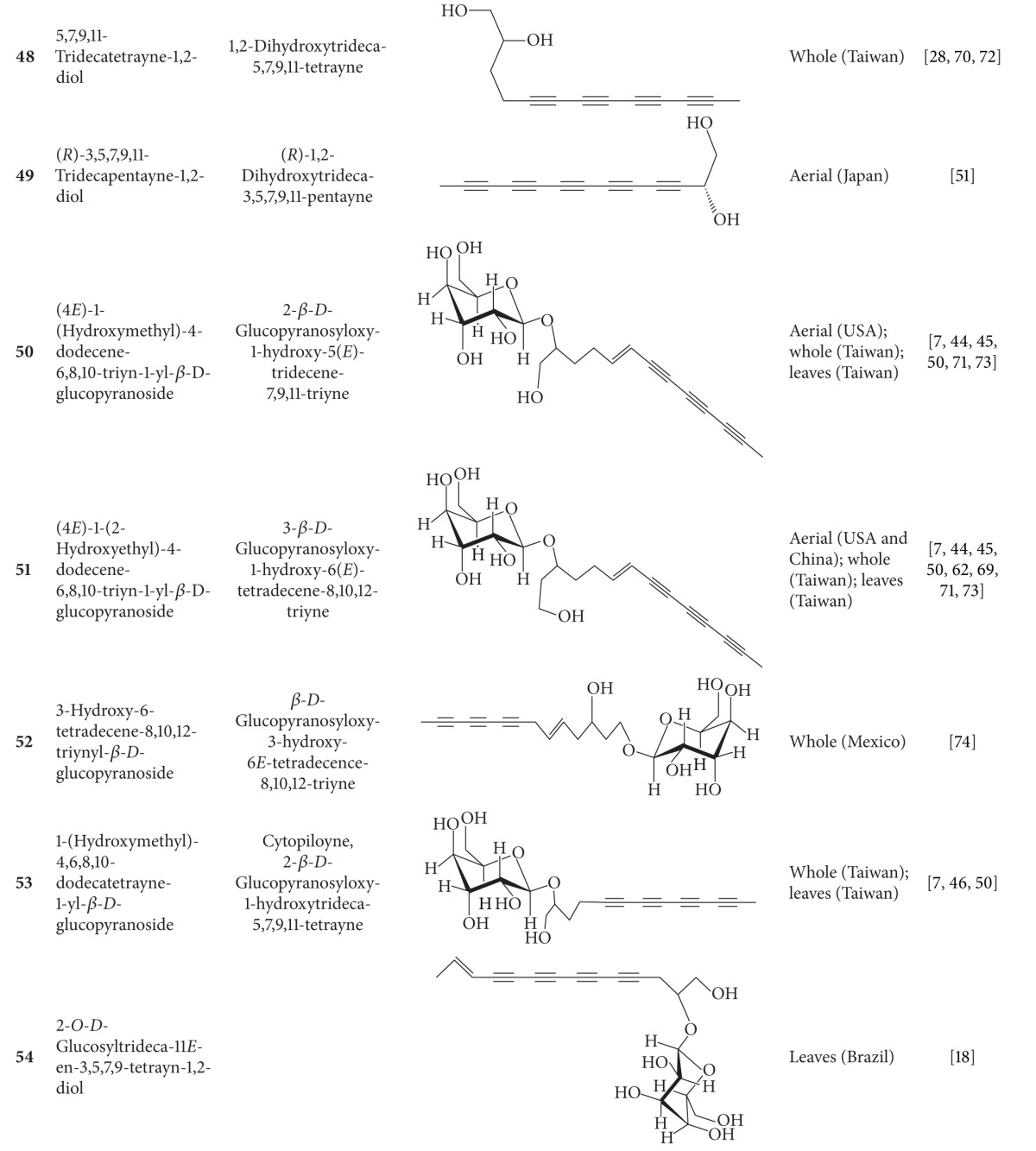 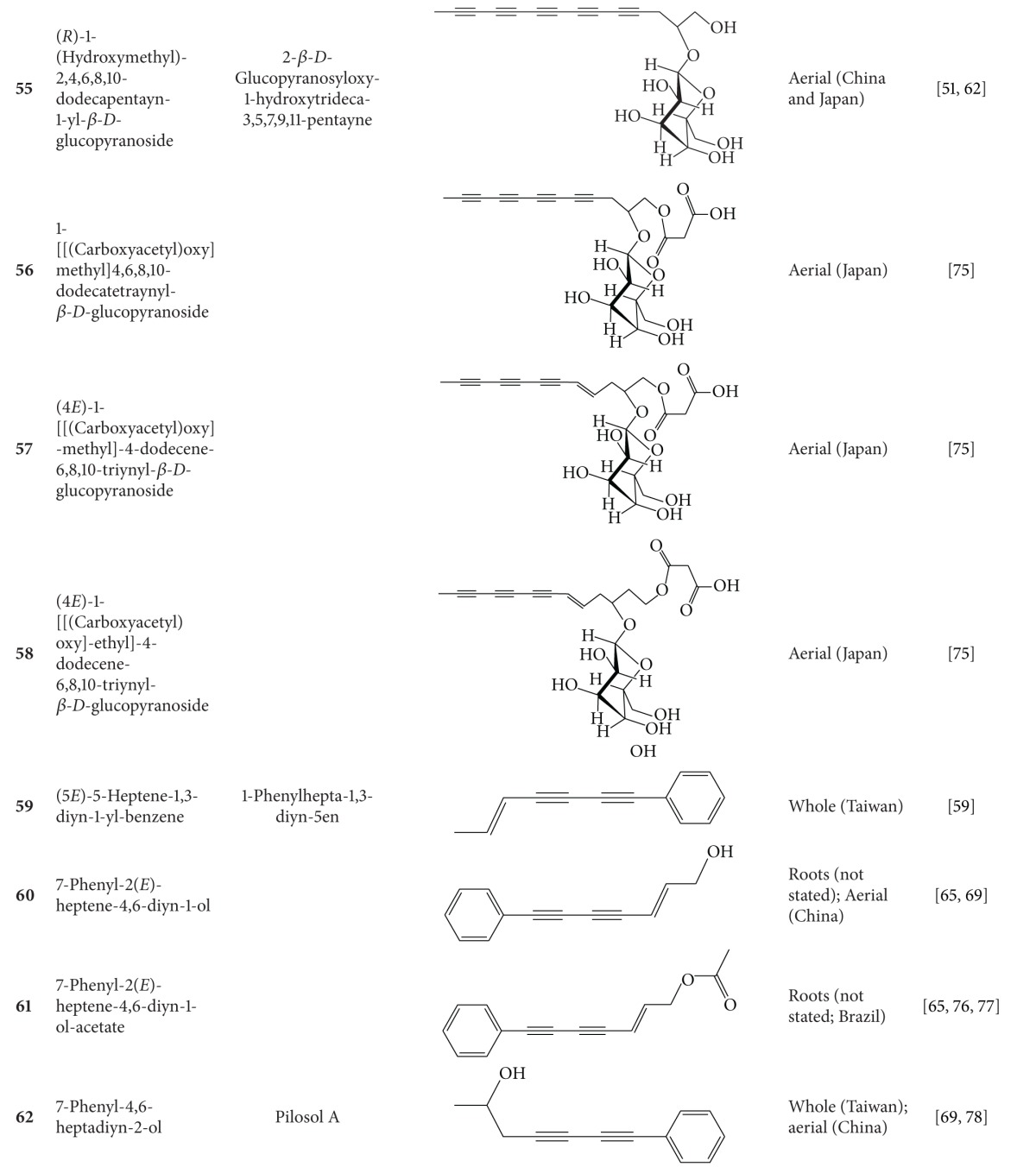 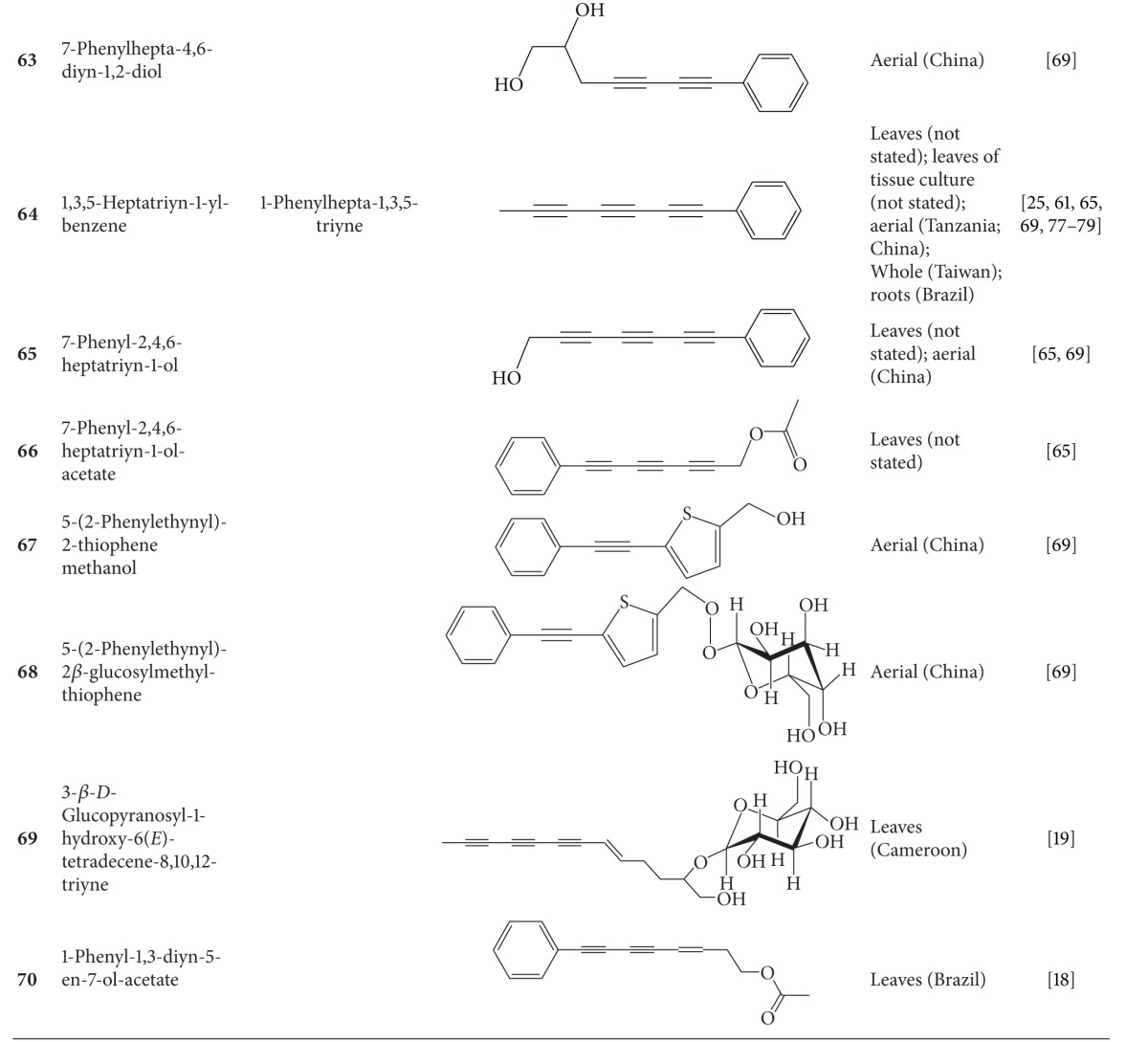

S.N. denotes serial number.

**Table 5:** Flavonoids isolated from *B. pilosa *[27].

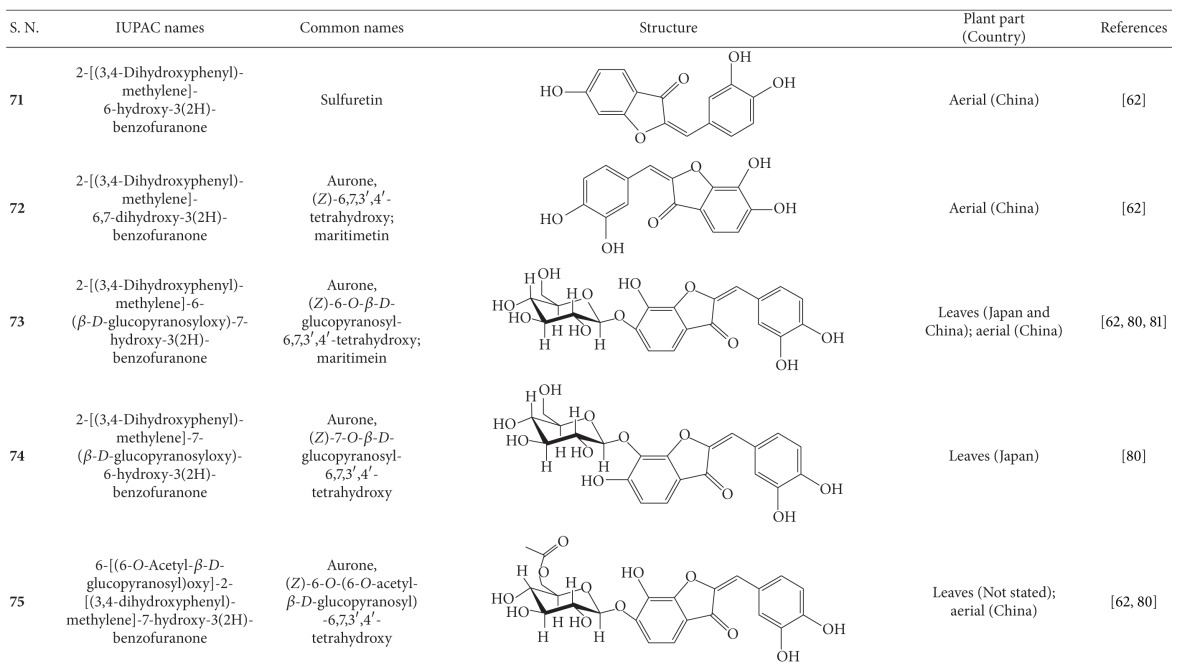 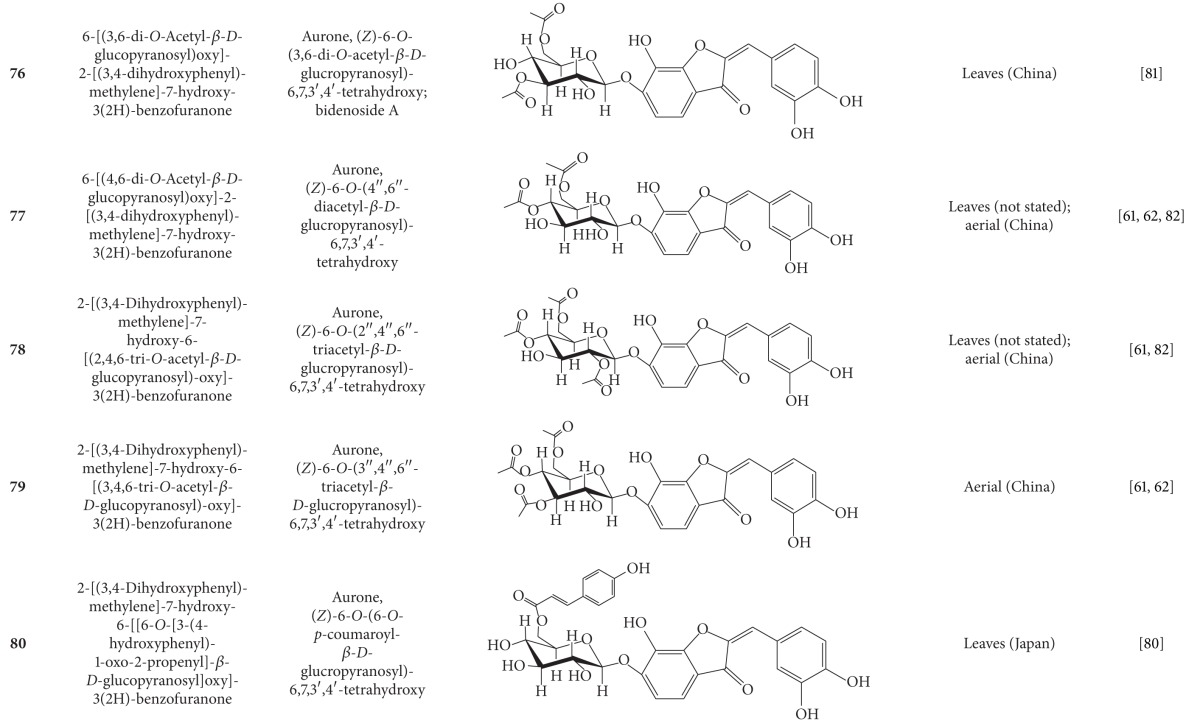 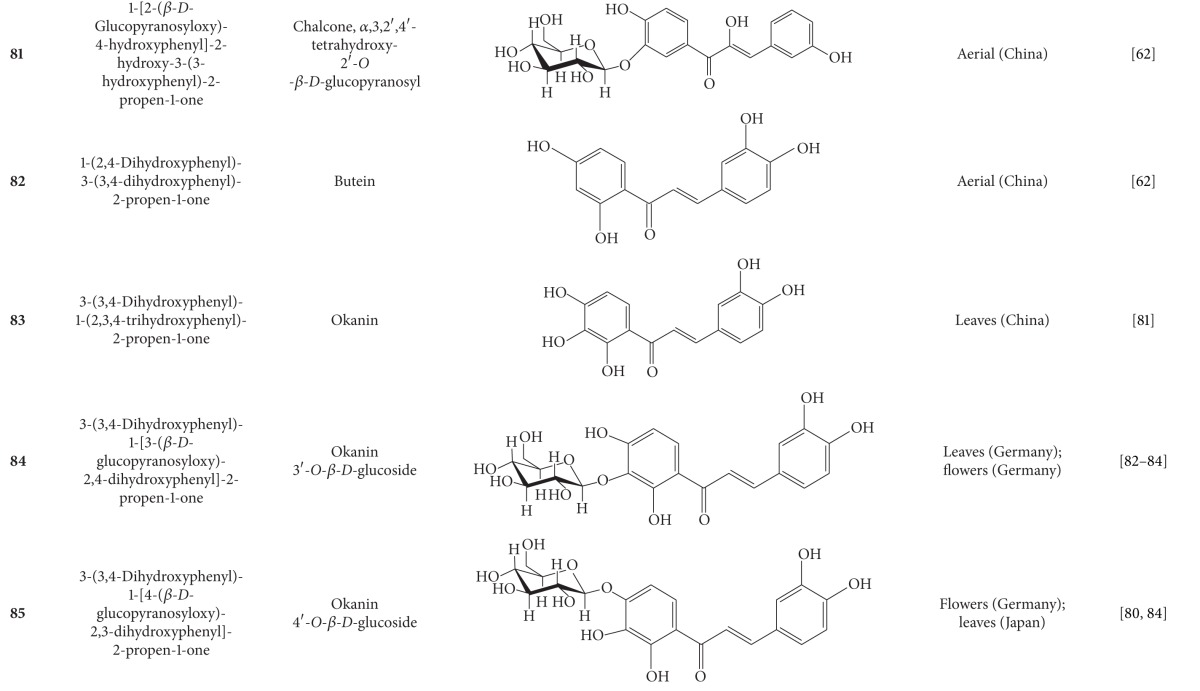 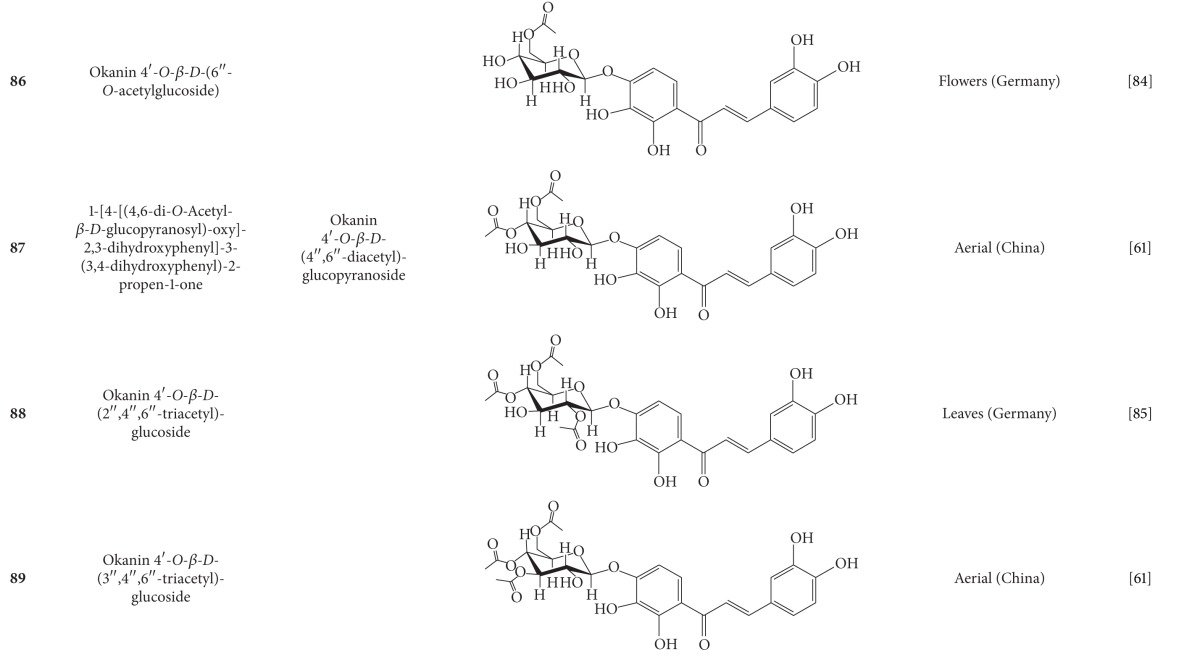 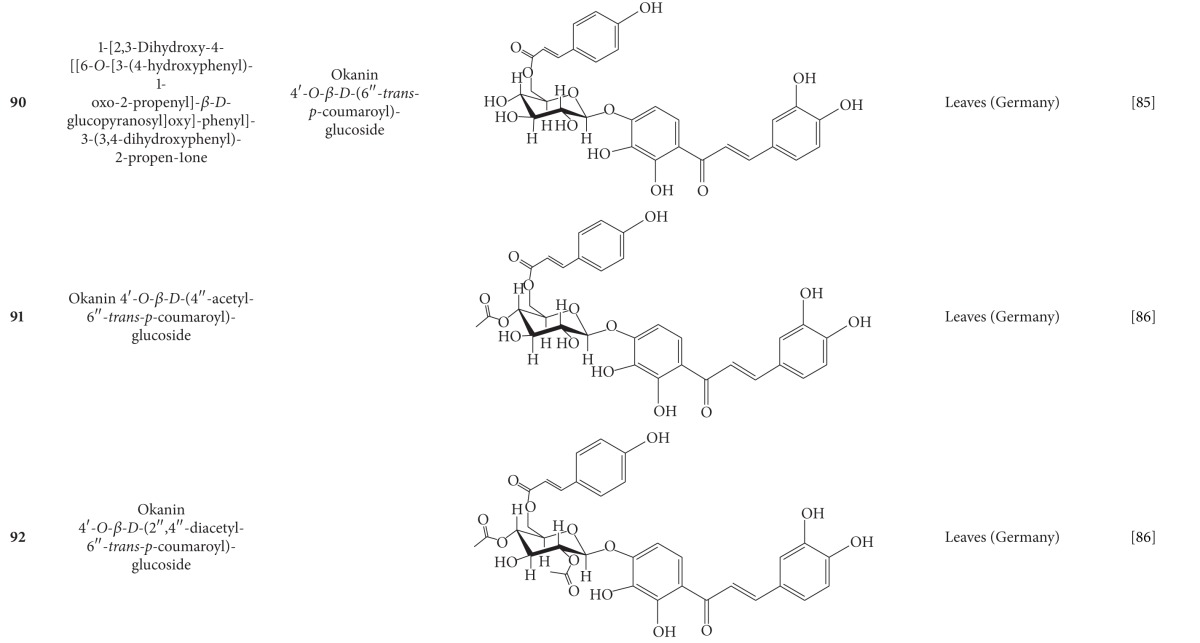 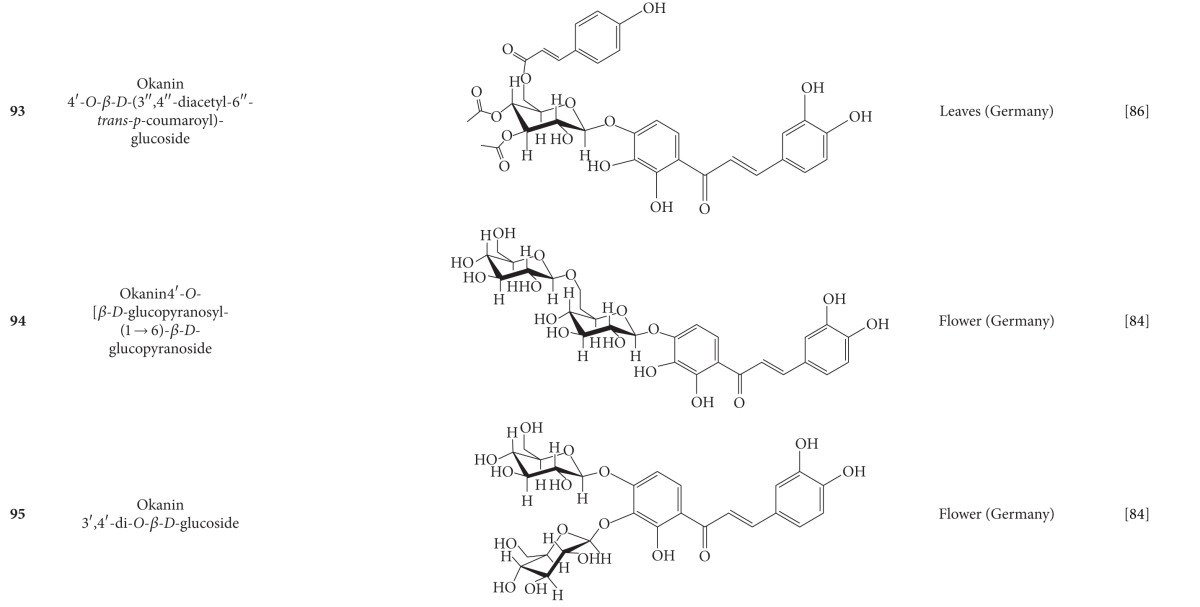 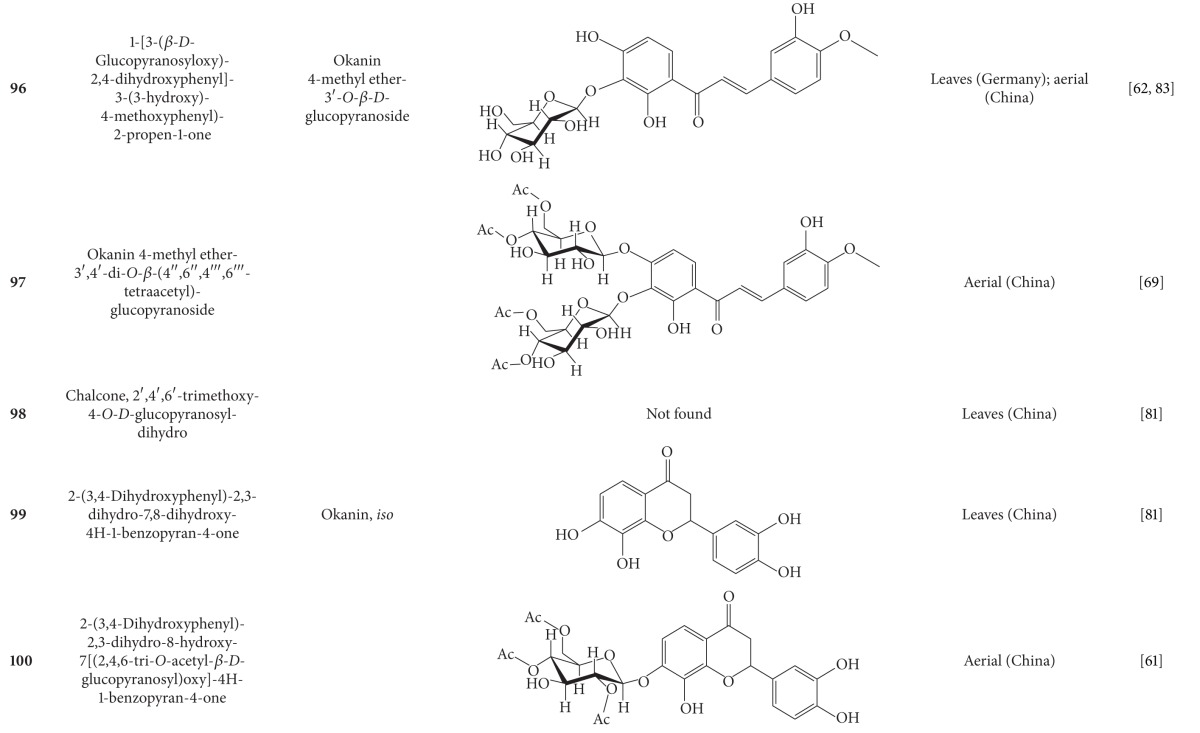 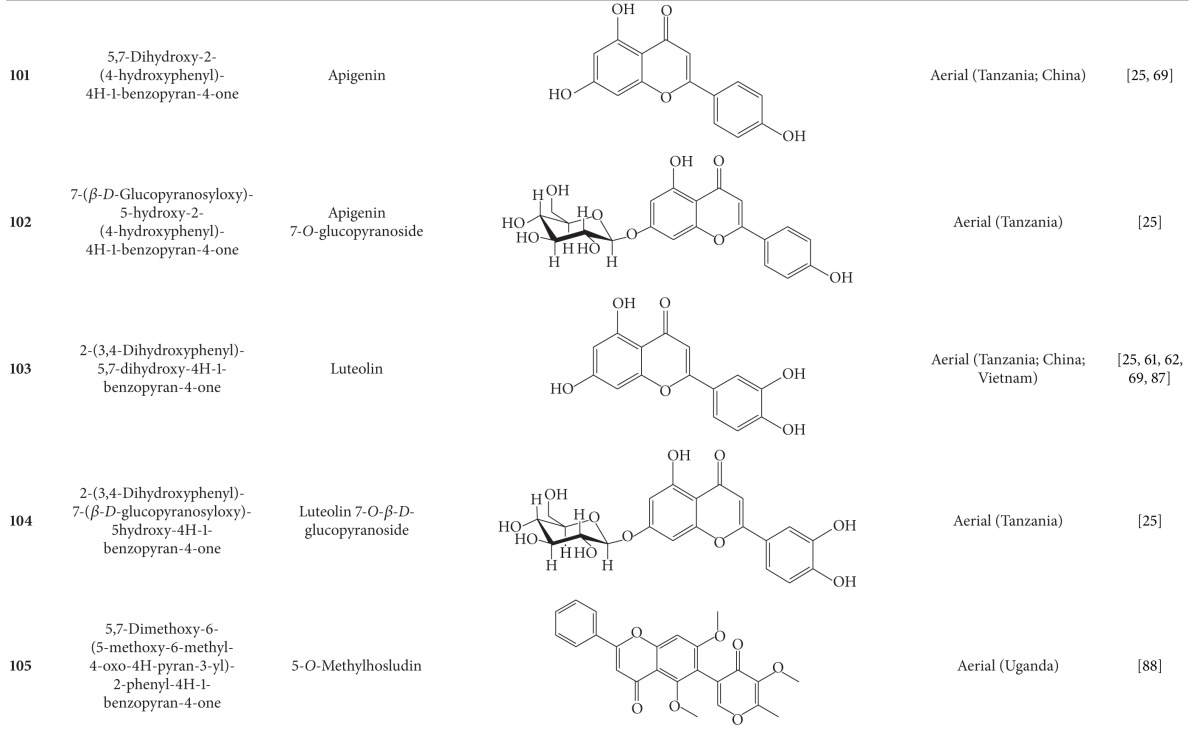 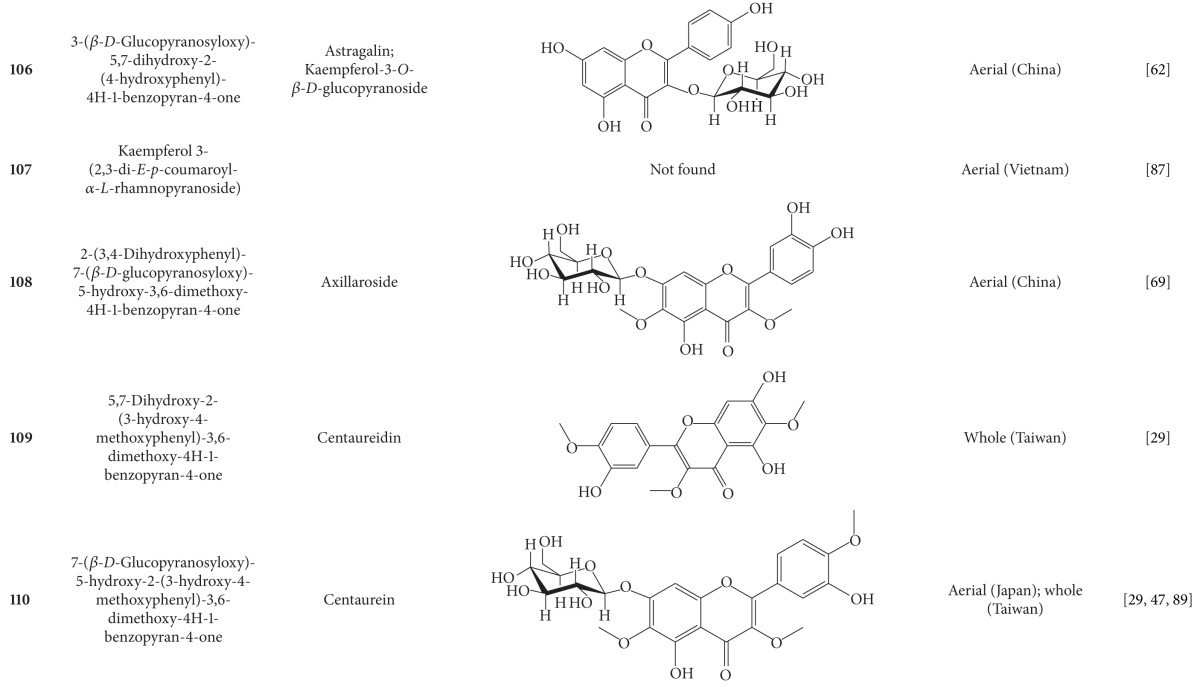 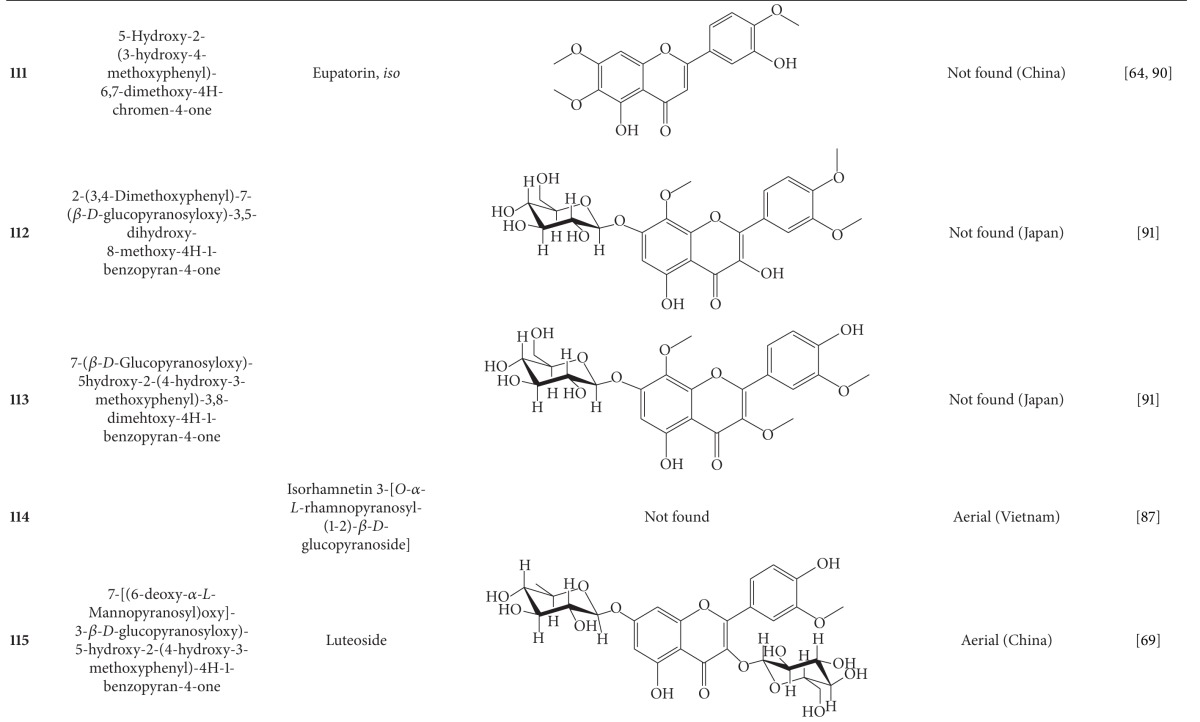 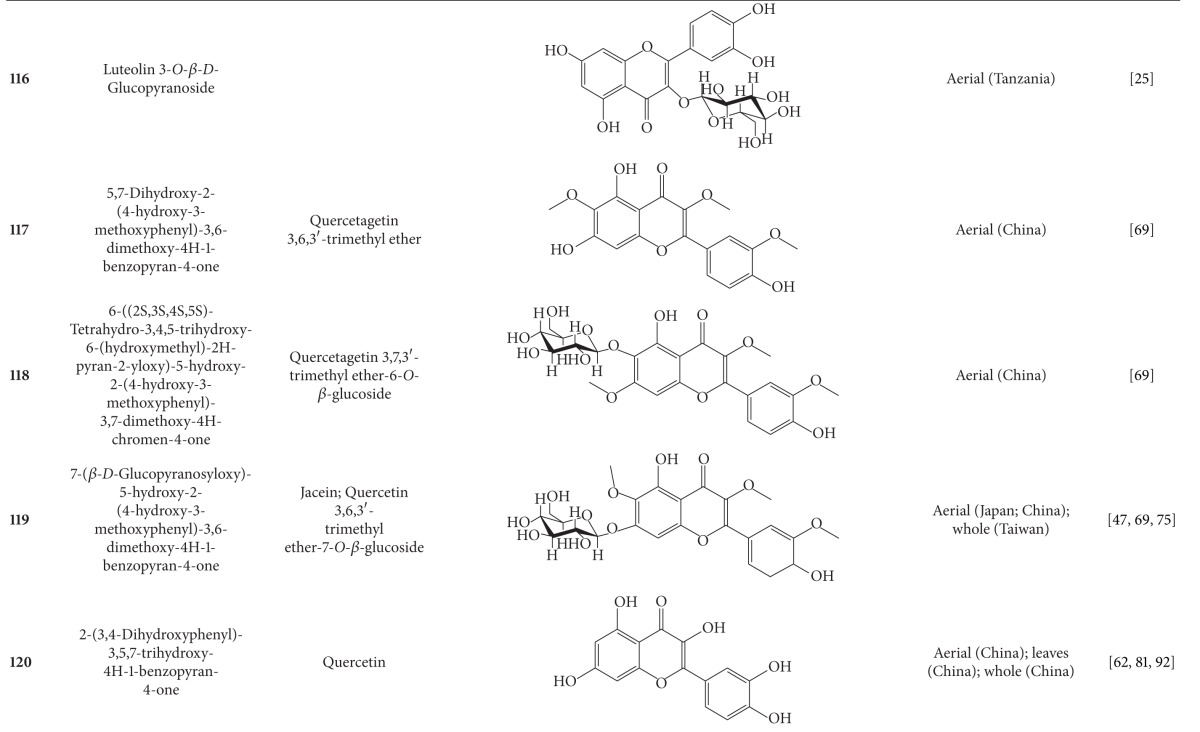 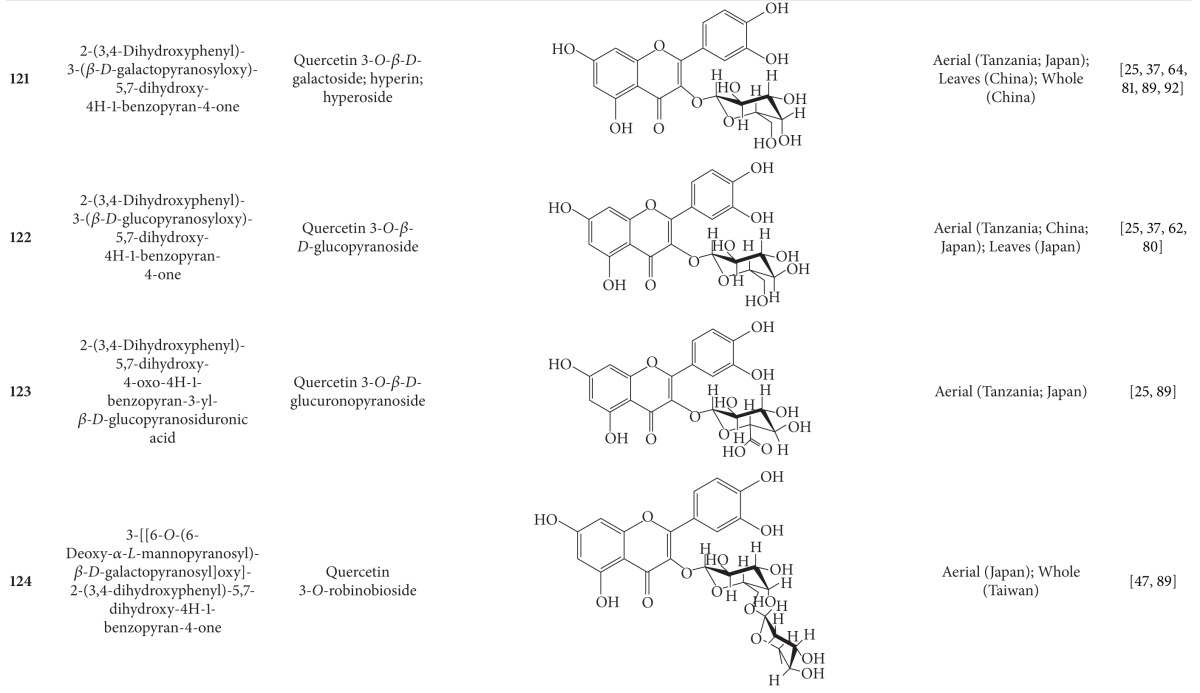 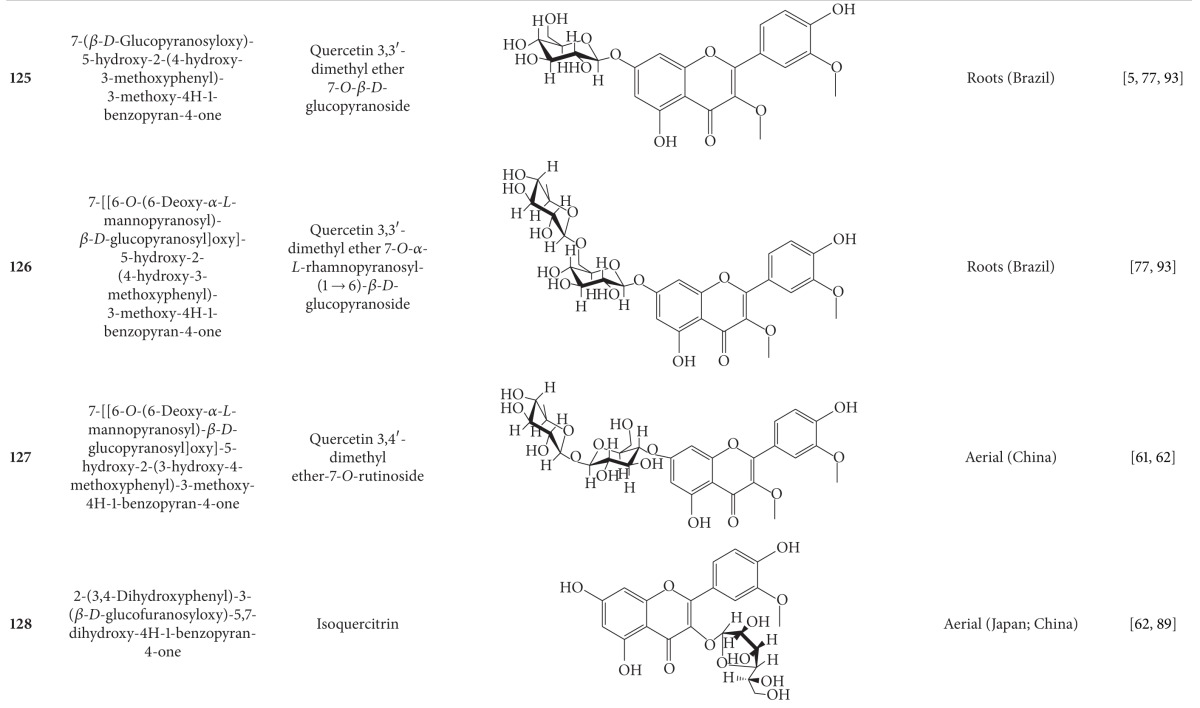 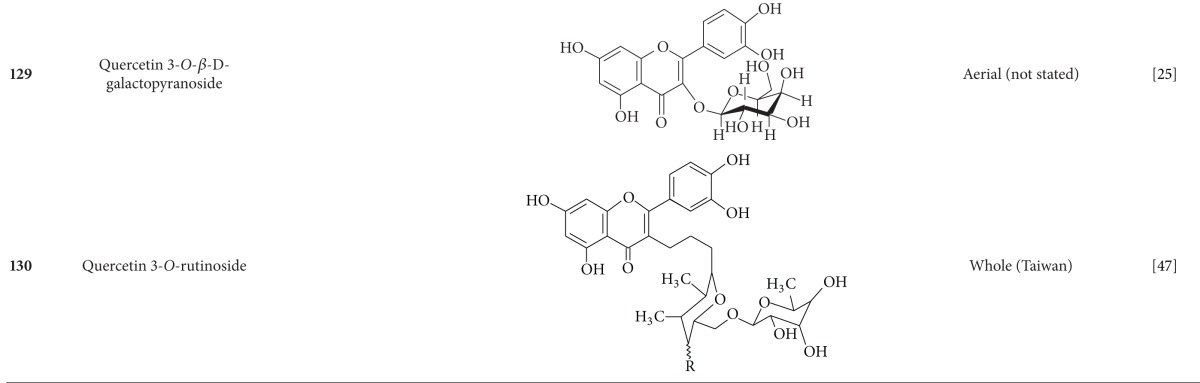

**Table 6:** Terpenoids isolated from *B. pilosa *[27].

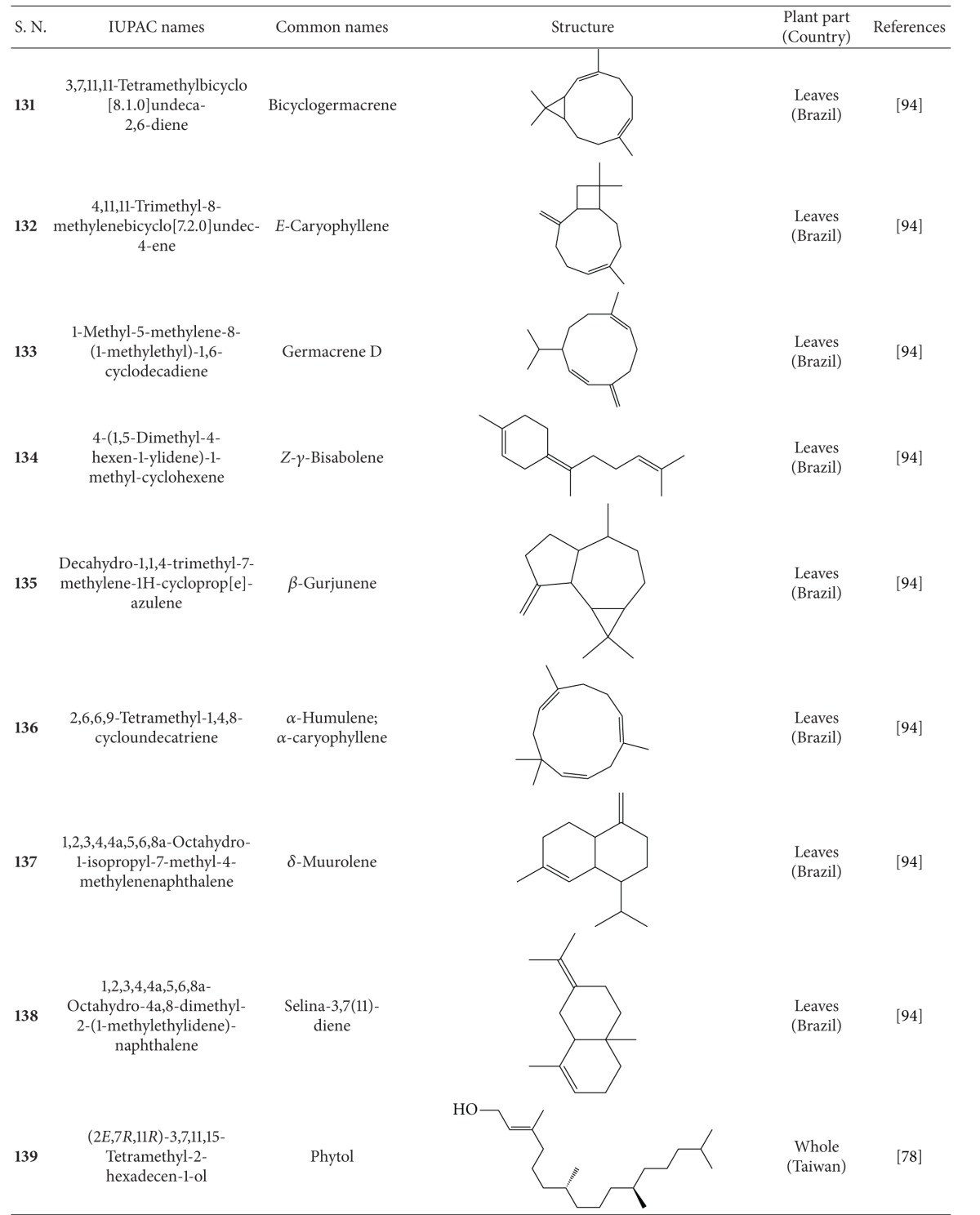 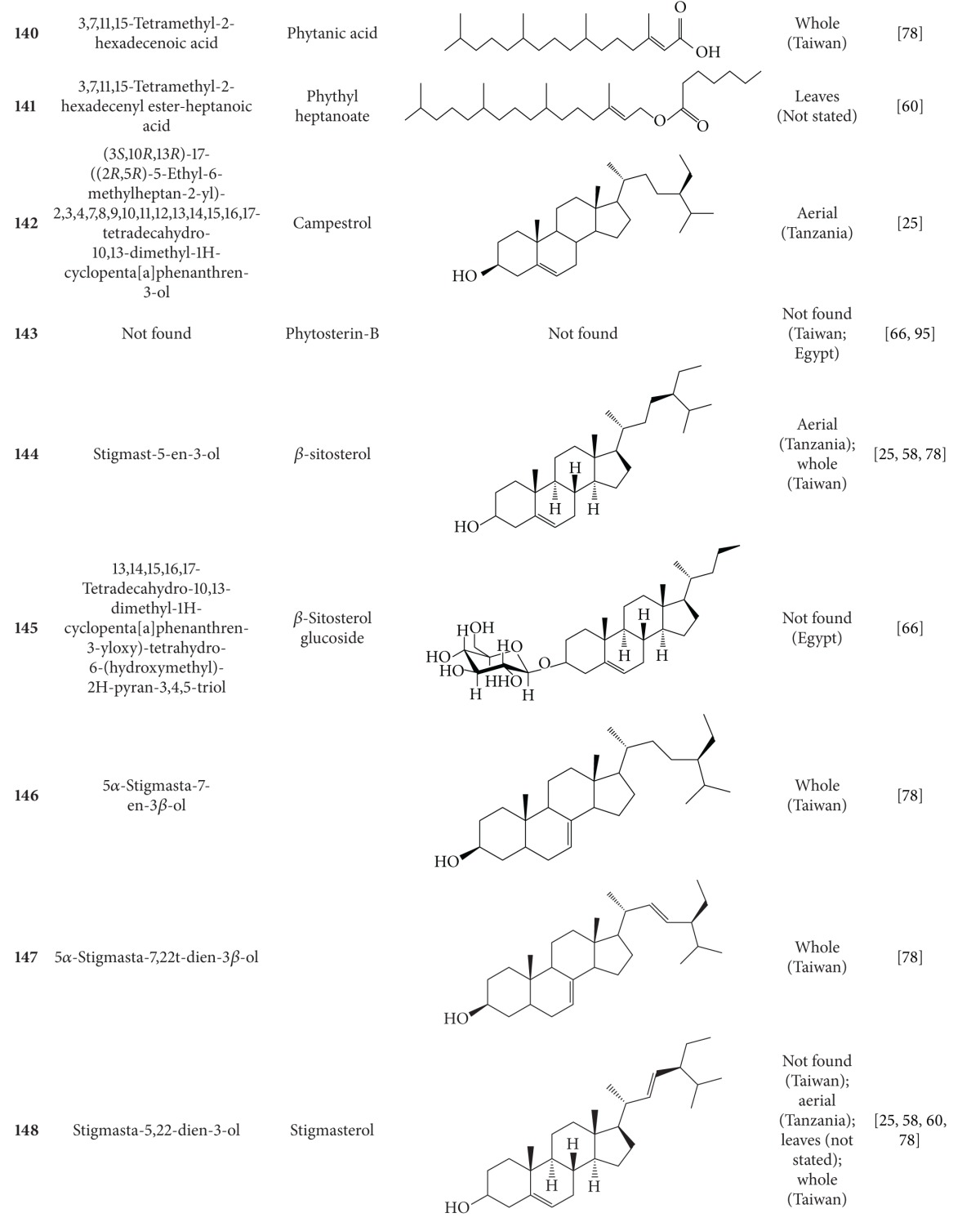 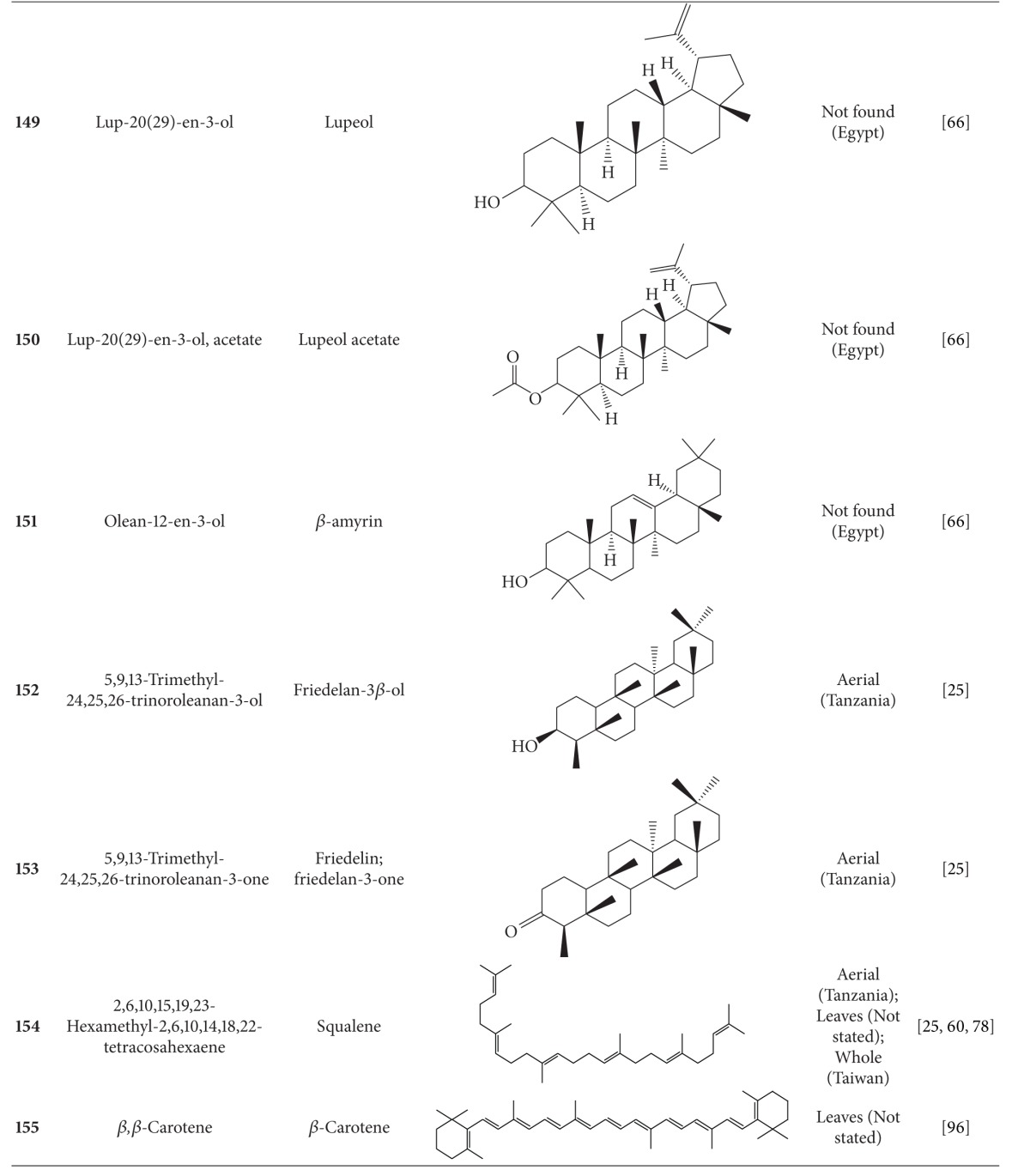

**Table 7:** Phenylpropanoids isolated from *B. pilosa *[27].

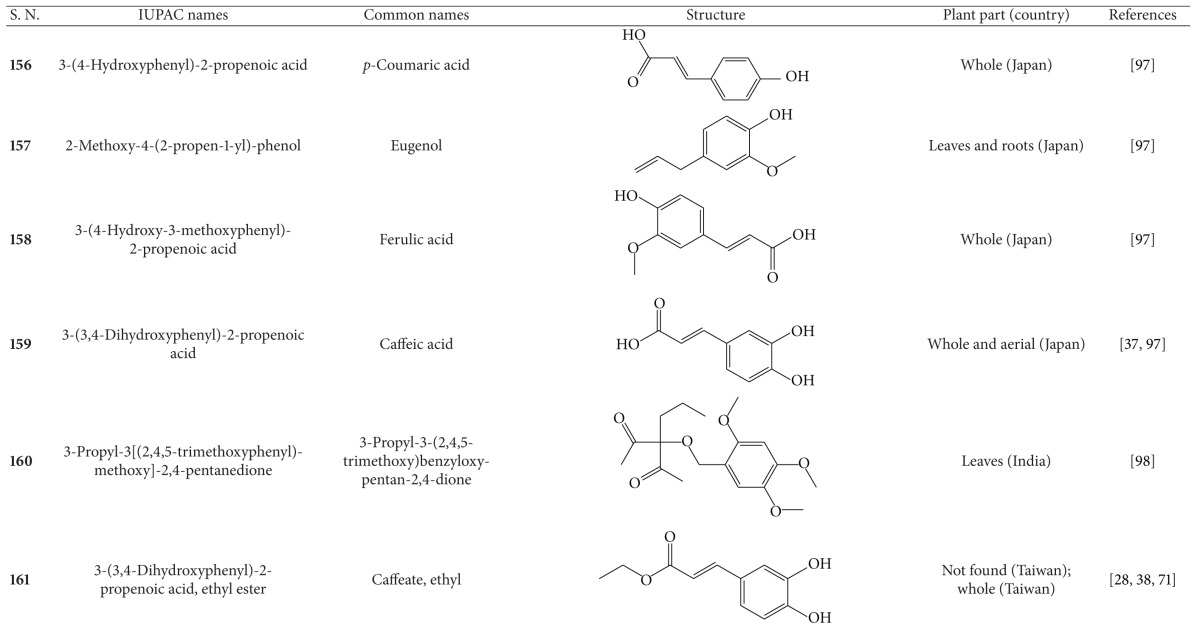 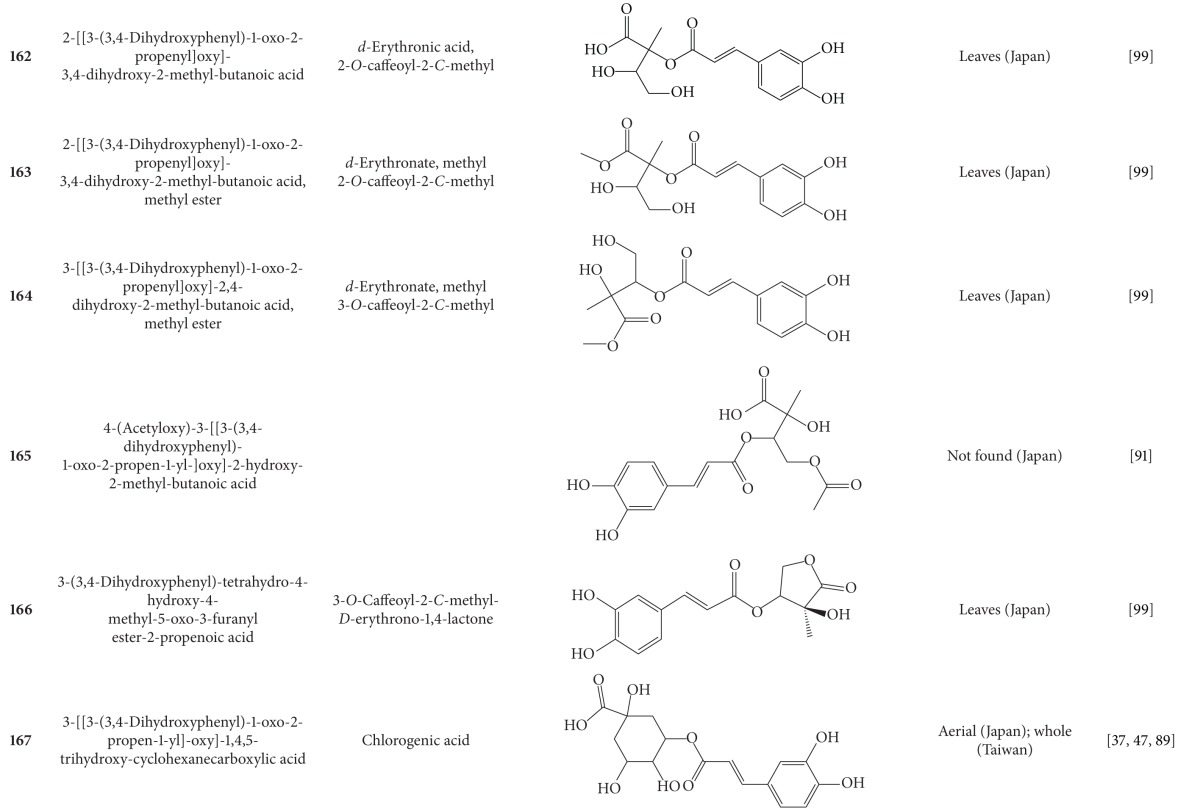 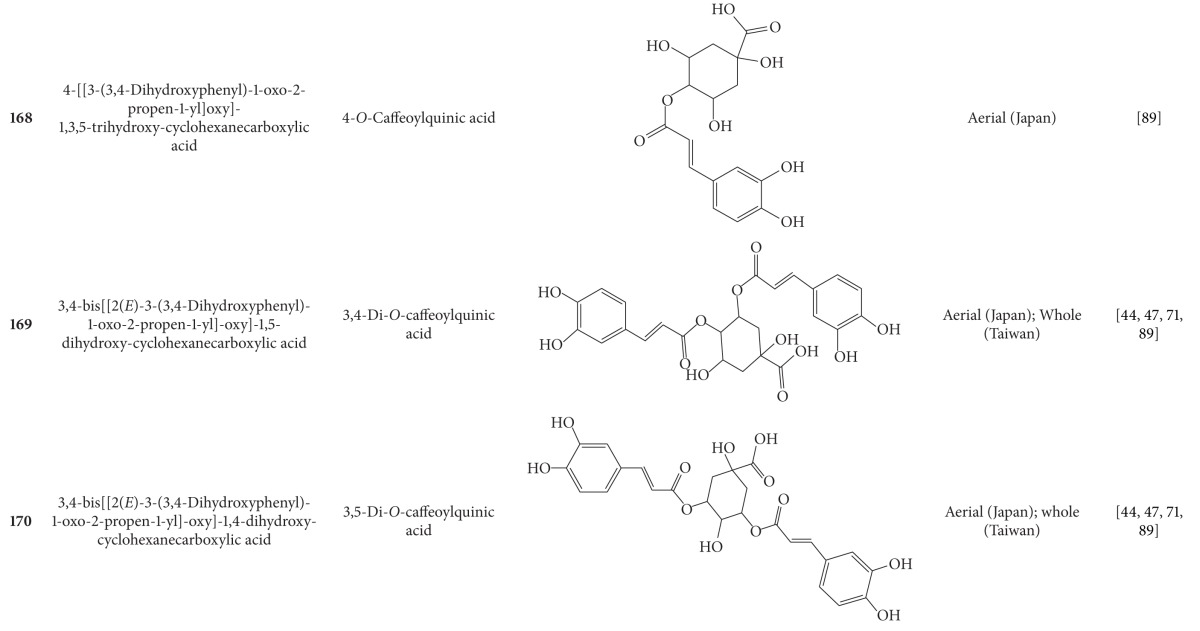 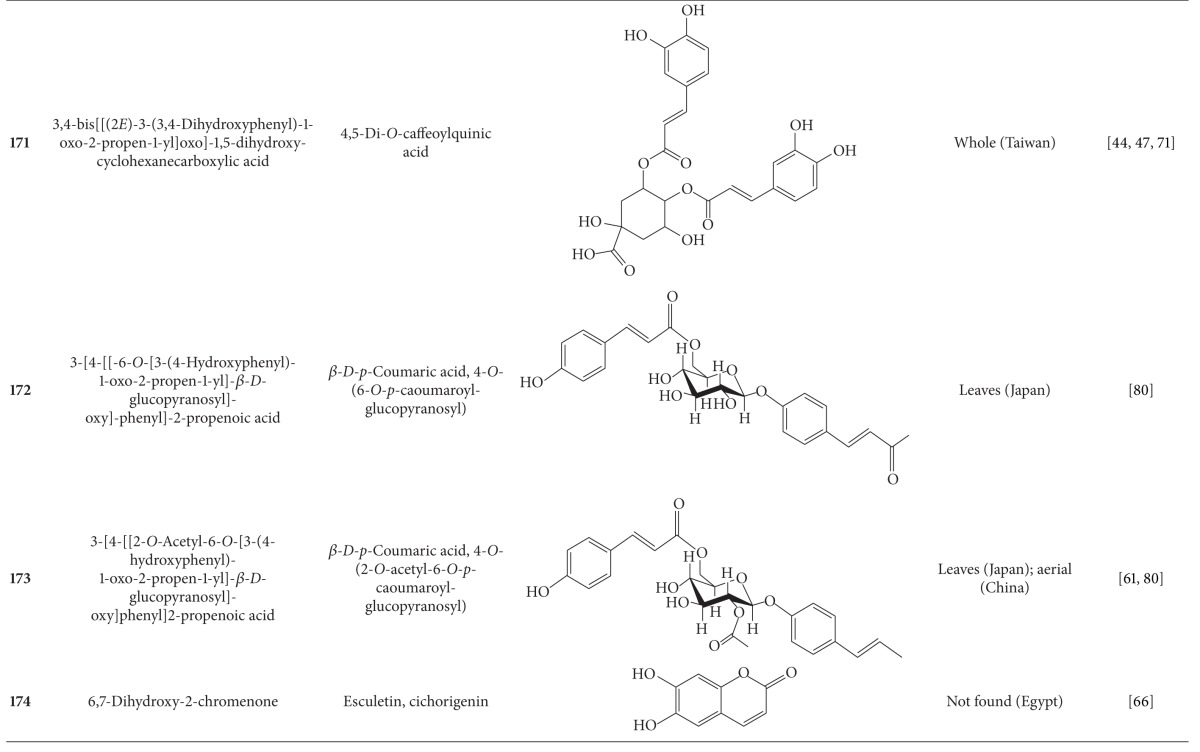

**Table 8:** Aromatic compounds isolated from *B. pilosa *[27].

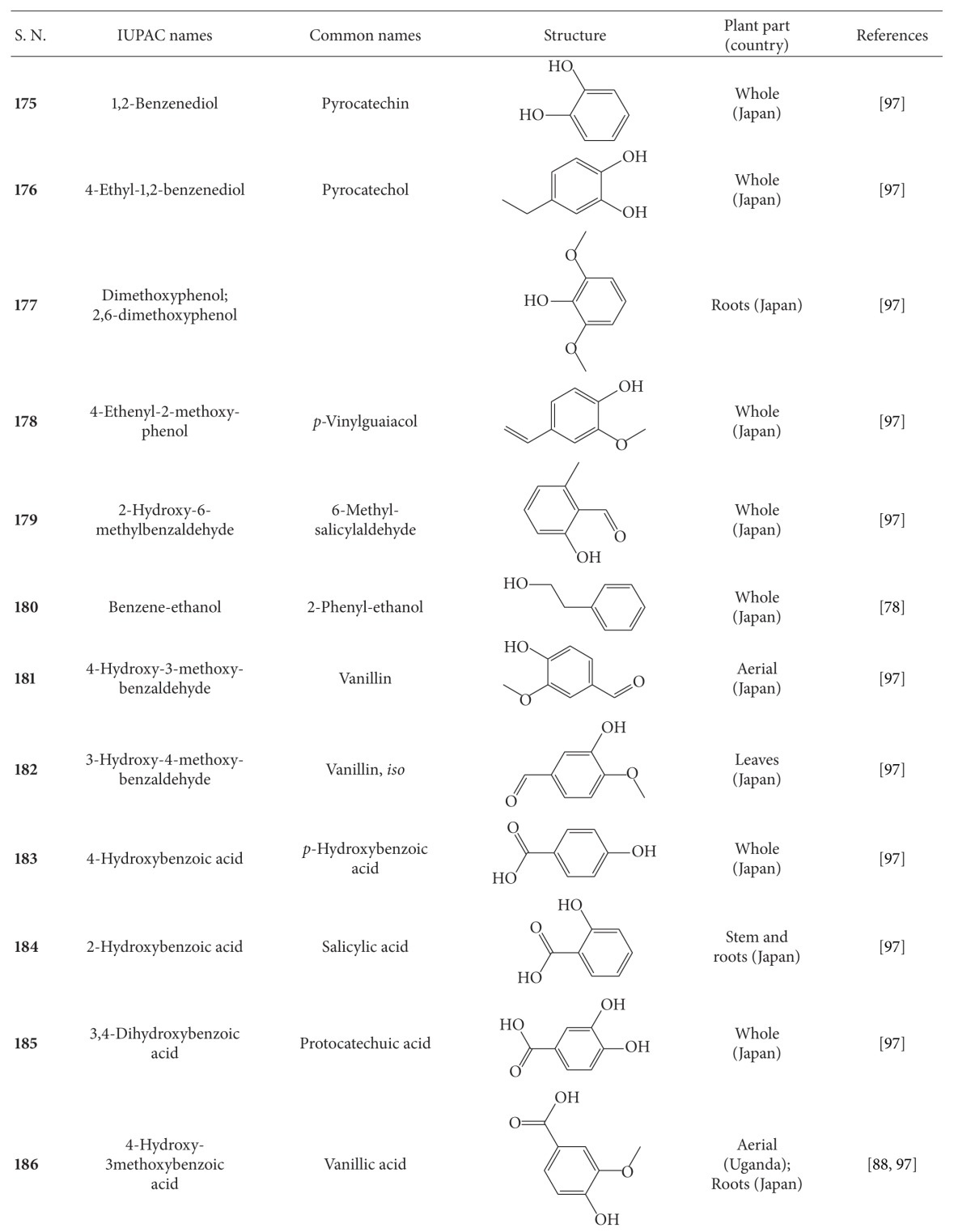 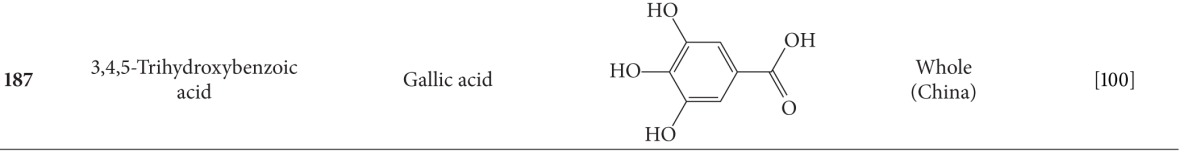

**Table 9:** Porphyrins isolated from *B. pilosa *[27].

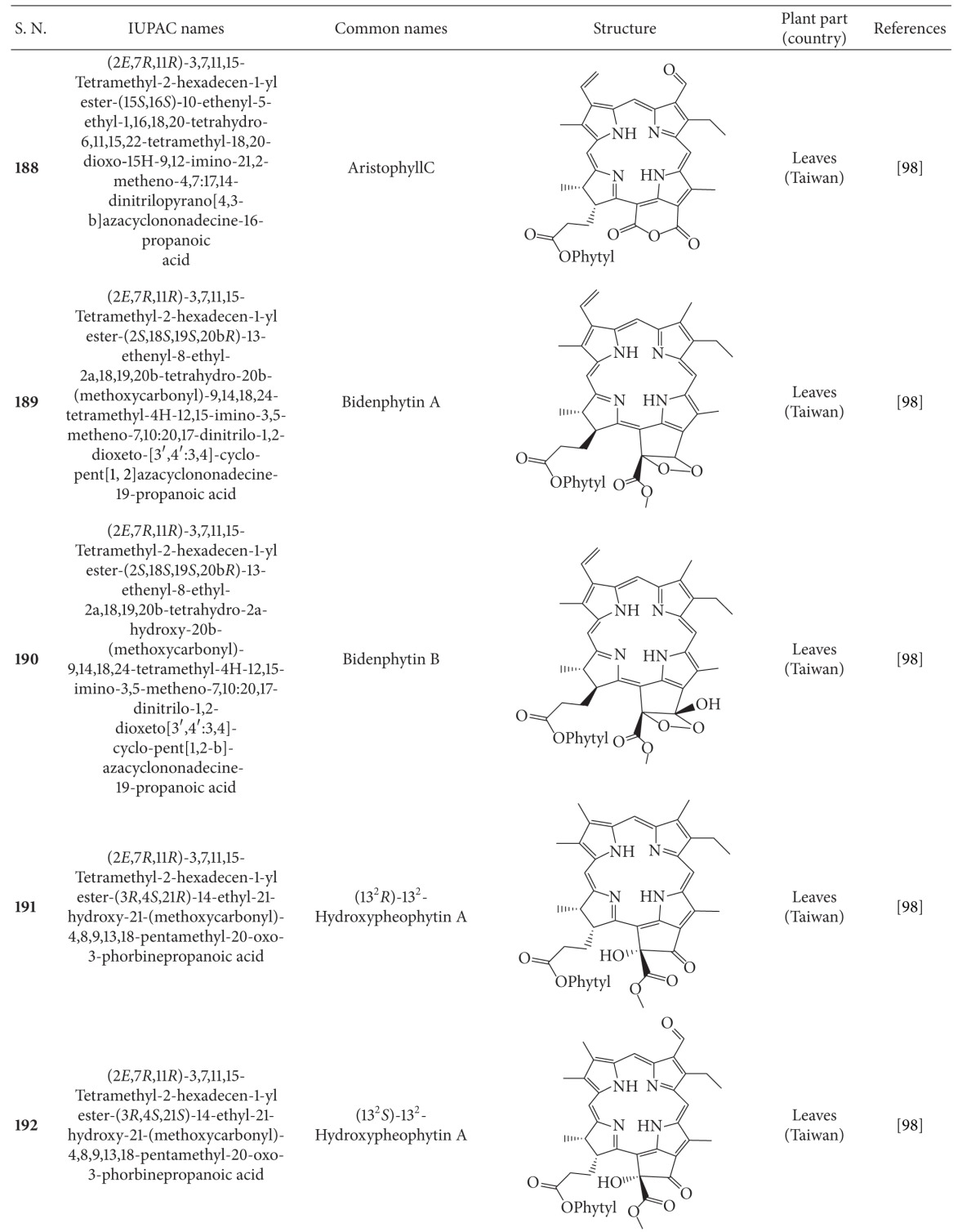 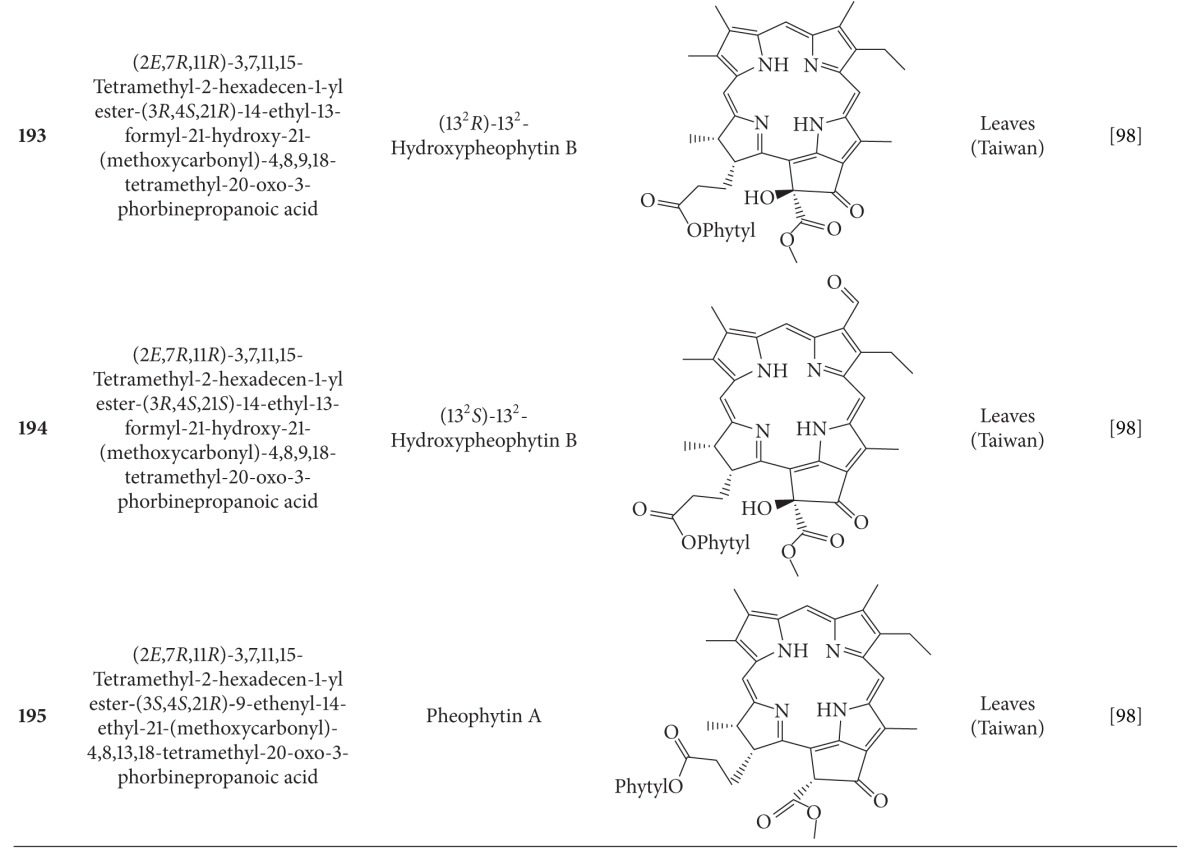

**Table 10:** Other compounds isolated from *B. pilosa *[27].

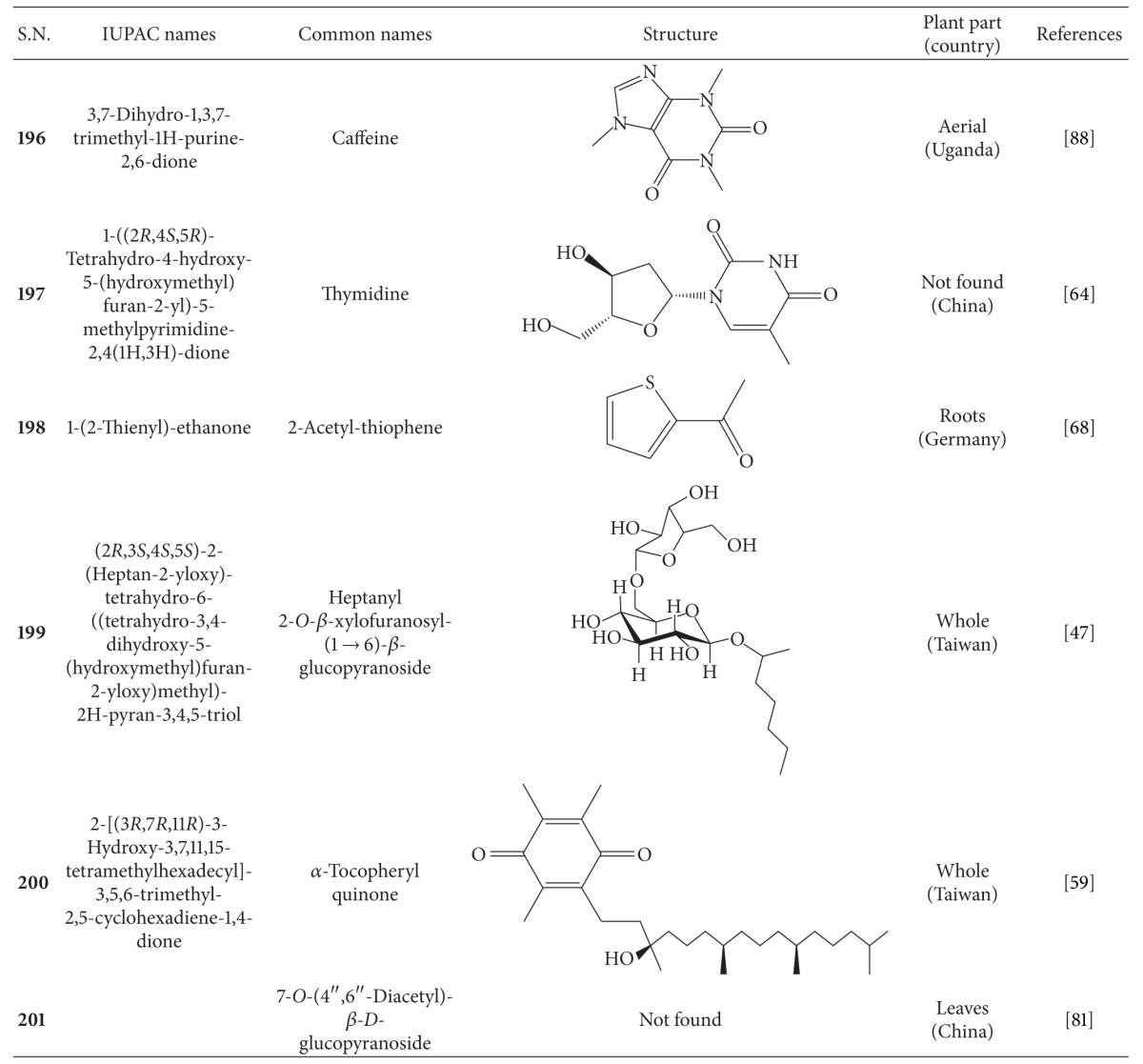

**Table 11:** Chemical constituents of *B. pilosa* and their biological activities.

S.N.	Name	Classification	Molecular formula	Biological activities
**109**	Centaureidin [52]	Flavonoid	C_18_H_16_O_8 _	Anti-listerial [29, 52] Cytotoxic [9]
**110**	Centaurein [52]	Flavonoid	C_24_H_26_O_13_	Anti-listerial [29, 52] Cytotoxic [9] Anti-viral [104]
**103**	Luteolin [105]	Flavonoid	C_15_H_10_O_6_	Anti-viral [106, 107] Cytotoxic [30] Anti-inflammatory [108] Anti-allergic [108]
**82**	Butein [109]	Flavonoid	C_15_H_12_O_5_	Anti-leishmanial [110] Cytotoxic [31]
**48**	1,2-Dihydroxytrideca-5,7,9,11-tetrayne [111]	Polyyne	C_13_H_12_O_2_	Anti-angiogeneic [111]
**46**	1,3-Dihyroxy-6(*E*)-tetradecene-8,10,12-triyne [111]	Polyyne	C_14_H_16_O_2_	Anti-angiogeneic [111]
**45**	1,2-Dihyroxy-5(*E*)-tridecene-7,9,11-triyne [112]	Polyyne	C_13_H_14_O_2_	Anti-angiogeneic [112] Anti-proliferative [112]
**64**	1-Phenylhepta-1,3,5-triyne [113]	Polyyne	C_13_H_8_	Anti-microbial [114] Anti-malarial [3] Cytotoxic [3] Antifungal [14]
**27**	Linoleic acid [32]	Fatty acid	C_18_H_32_O_2_	Anti-viral (100) Cytotoxic [23]
**161**	Ethyl caffeate [38]	Phenylpropanoid	C_11_H_12_O_4_	Anti-inflammatory [38]
**54**	2-*O*-*β*-Glucosyltrideca-11(*E*)-en-3,5,7,9-tetrayn-1,2-diol [17]	Polyyne	C_19_H_20_O_7_	Immunosuppressive and Anti-inflammatory [17]
**53**	2-*β*-*D*-Glucopyranosyloxy-1-hydroxytrideca-5,7,9,11-tetrayne [50]	Polyyne	C_19_H_22_O_7_	Anti-diabetic [115] Anti-inflammatory [50]
**69**	3-*β*-*D*-Glucopyranosyl-1-hydroxy-6(*E*)-tetradecene-8,10,12-triyne [19]	Polyyne	C_20_H_26_O_7_	Anti-diabetic [19] Anti-inflammatory [55]
**50**	2-*β*-*D*-Glucopyranosyloxy-1-hydroxy-5(*E*)-tridecene-7,9,11-triyne [19]	Polyyne	C_19_H_24_O_7_	Anti-diabetic [19] Anti-inflammatory [55] Anti-malarial and antibacterial [51]
**129**	Quercetin 3-*O*-*β*-*D*-galactopyranoside [25]	Flavonoid	C_21_H_20_O_12_	Anti-inflammatory [116]
**170**	3,5-Di-*O*-caffeoylquinic acid [55]	Phenylpropanoid	C_25_H_24_O_12_	Anti-viral [78] Antioxidant [47]
**171**	4,5-Di-*O*-caffeoylquinic acid [55]	Phenylpropanoid	C_25_H_24_O_12_	Anti-viral [78] Antioxidant [47]
**169**	3,4-Di-*O*-caffeoylquinic acid [55]	Phenylpropanoid	C_25_H_24_O_12_	Anti-viral [78] Antioxidant [47]
**126**	Quercetin 3,3′-dimethyl ether 7-*O*-*α*-*L*-rhamnopyranosyl-(1→6)-*β*-*D*-glucopyranoside [117]	Flavonoid	C_21_H_20_O_11_	Anti-malarial [118]
**125**	Quercetin 3,3′-dimethyl ether-7-*O*-*β*-*D*-glucopyranoside [117]	Flavonoid	C_21_H_20_O_12_	Anti-malarial [118]
**70**	1-Phenyl-1,3-diyn-5-en-7-ol-acetate [18]	Polyyne	C_15_H_12_O_2_	Anti-malarial [18]
**199**	Heptanyl 2-*O*-*β*-xylofuranosyl-(1→6)-*β*-glucopyranoside [47]	Miscellaneous	C_18_H_44_O_10_	Antioxidant [47]
**124**	3-*O*-Rabinobioside [47]	Saccharide	C_27_H_38_O_15_	Antioxidant [47]
**130**	Quercetin 3-*O*-rutinoside [47]	Flavonoid	C_27_H_38_O_15_	Antioxidant [47]
**167**	Chlorogenic acid [47]	Phenolic	C_16_H_26_O_9_	Antioxidant [47]
**119**	Jacein [47]	Flavonoid	C_24_H_26_O_13_	Antioxidant [47]
**49**	(*R*)-1,2-dihydroxytrideca-3,5,7,9,11-pentayne [51]	Polyyne	C_13_H_8_O_2_	Anti-malarial and Antibacterial [51]

**Table 12:** Structure and activity relationship studies of ethyl caffeate using* in vitro* NF-*κ*B/DNA binding assays [38].

Compound	Concentration (*μ*M)	NF-*κ*B/ DNA binding
Ethyl caffeate	50	100% inhibition
Ethyl 3,4-dihydroxyhydrocinnamate	100	100% inhibition
Catechol	400	100% inhibition
Ethyl cinnamate	400	No inhibition

**Table 13:** Apoptosis in cocultures of T cells and pancreatic *β* cells [46].

Cell/Medium	% Apoptosis and necrosis
CD4^+^ T cells/control medium	<4
CD4^+^ T cells/with PBS-treated *β* cells of NOD-SCID mice	2
CD4^+^ T cells/with cytopiloyne-treated *β* cells	18
CD4^+^ T cells/with cytopiloyne-treated *β* cells in the presence of *α*-FasL antibody	7
CD8^+^ T cells/control medium	4
CD4^+^ T cells/with PBS-treated *β* cells of NOD-SCID mice	4
CD8^+^ T cells/with cytopiloyne-treated *β* cells	4
CD8^+^ T cells/with cytopiloyne-treated *β* cells in the presence of *α*-FasL antibody	4

**Table 14:** Radical scavenging activities of *B. pilosa *extracts [47].

Extracts/control	DPPH assay, IC_50_ (*μ*g/mL)	NBT/hypoxanthine superoxide assay, IC_50_ (*μ*g/mL)
Quercetin	1.98	1.5
Ascorbic acid	6.34	Not determined
*α*-tocopherol	8.97	Not determined
Ethyl acetate extract	13.83	59.7
Butanol extract	16.69	11.4
Water extract	>100	>100

**Table 15:** Radical scavenging activity of secondary metabolites from *B. pilosa* [47].

Metabolite (Table 11)/Control	DPPH assay, IC_50 _(*μ*g/mL)
**199**	Not determined
**124**	5.3
**130**	6.8
**167**	10.5
**169**	3.3
**171**	3.8
**119**	Not determined
**110**	Not determined
Quercetin	2.56
Caffeic acid	8.90

**Table 16:** Antioxidant activity of the essential oils and water extracts from *B. pilosa *[53].

Extract	IC_50_ (*μ*g/mL)
Leaf essential oils	47
Flower essential oils	50
Leaf extract	61
Flower extract	172

**Table 17:** Antibacterial activity of essential oils and flower extracts from *B. pilosa *[53].

Strain	Mean zone of inhibition (mm)			
	Leaf essential oil	Flower essential oil	Leaf extract	Flower extract
*Micrococcus flavus *	12.7 ± 0.3	8.7 ± 0.3	10.2 ± 0.2	10.8 ± 0.3
*Bacillus subtilis *	17.3 ± 1.9	11.7 ± 0.2	10.9 ± 0.2	10.3 ± 0.2
*Bacillus cereus *	19.0 ± 1.4	11.2 ± 0.3	11.8 ± 0.4	18.5 ± 1.0
*Bacillus pumilus *	12.3 ± 0.7	10.8 ± 0.2	10.5 ± 0.4	7.7 ± 0.2
*Escherichia coli *	13.7 ± 0.4	20.3 ± 0.7	10.2 ± 1.1	14.0 ± 1.3
*Pseudomonas ovalis *	12.5 ± 0.8	13.7 ± 1.5	10.2 ± 0.6	12.5 ± 0.6

**Table 18:** Antibacterial activity of root extracts from *B. pilosa* [54].

Strain	MIC_50_ (mg/mL)	
	Methanol extract	Acetone extract
*Bacillus cereus *	10	—
*Escherichia coli *	5	5
*Klebsilla pneumonia *	5	10
*Micrococcus kristinae *	10	—
*Pseudomonas aeruginosa *	10	10
*Staphylococcus aureus *	5	10
*Sraphylococcus epidermidis *	5	5
*Serratia marcescens *	10	—
*Shigelea flexneri *	10	—
*Streptococcus faecalis *	10	—

**Table 19:** Antibacterial activity of *B. pilosa* of compound **29** [51].

Strain	MIC_50 _(*μ*g/mL)
*Escherichia coli* NIHJ	1
*Escherichia coli* ATCC25922	1
*Klebsiella pneumoniae* ATCC700603	128
*Serratia marcescens* ATCC13880	16
*Pseudomonas aeruginosa* ATCC27853	8
*Staphylococcus aureus* FDA209P	0.5
*Staphylococcus aureus* ATCC29213	0.25
*Staphylococcus aureus* N315 (MRSA)	0.5
*Enterococcus faecalis* ATCC29212	2
*Enterococcus faecalis* NCTC12201 (VRE)	1
*Bacillus subtilis* ATCC6633	0.5
*Candida albicans* ATCC10231	0.25

**Table 20:** Antifungal activity of *B. pilosa* [53].

Part/extract		Concentration (ppm)	Strain, % Inhibition		
			*Cortiicum rolfsii *	*Fusarium solani *	*Fusarium oxysporum *
Leaves	Essential oils	100	85.7 ± 0.9	68.2 ± 0	74.5 ± 1.7
		250	96.0 ± 0.8	77.9 ± 1.8	87.9 ± 0.4
	Aqueous Extracts	100	44.6 ± 1.7	60.5 ± 2.1	71.6 ± 0.7
		250	94.2 ± 0.3	68.9 ± 0.7	82.4 ± 1.9

Flowers	Essential oils	100	60.4 ± 0.9	89.2 ± 0.4	86.9 ± 0.5
		250	89.4 ± 1.2	98.0 ± 0.3	94.9 ± 0.6
	Aqueous Extracts	100	33.1 ± 1.1	71.4 ± 0.7	57.3 ± 2.2
		250	66.1 ± 1.4	91.2 ± 0	90.0 ± 0.7

**Table 21:** Antifungal activity of *B. pilosa* root extracts [54].

Strain	LC_50_ (mg/mL)		
	Acetone extracts	Methanol extracts	Water extracts
*Aspergillus niger *	0.14	0.06	0.07
*Aspergillus flavus *	10.91	6.58	0
*Penicillium notatum *	0.05	0.05	0.05

## References

[1] Shen T, Li GH, Wang XN, Lou HX The genus *Commiphora*: a review of its traditional uses, phytochemistry and pharmacology. *Journal of Ethnopharmacology*.

[2] Long C, Sauleau P, David B (2003). Bioactive flavonoids of **Tanacetum parthenium** revisited. *Phytochemistry*.

[3] Karis PO, Ryding O (1994). *Asteraceae Cladistics and Classification*.

[4] Pozharitskaya ON, Shikov AN, Makarova MN (2010). Anti-inflammatory activity of a HPLC-fingerprinted aqueous infusion of aerial part of **Bidens tripartita** L. *Phytomedicine*.

[5] Oliveira FQ, Andrade-Neto V, Krettli AU, Brandão MGL (2004). New evidences of antimalarial activity of **Bidens pilosa** roots extract correlated with polyacetylene and flavonoids. *Journal of Ethnopharmacology*.

[6] Agriculture USDA Plants database. http://www.nrcs.usda.gov/wps/portal/nrcs/site/national/home.

[7] Chien SC, Young PH, Hsu YJ (2009). Anti-diabetic properties of three common *Bidens pilosa* variants in Taiwan. *Phytochemistry*.

[8] Alcaraz MJ, Jimenez MJ (1988). Flavonoids as anti-inflammatory agents. *Fitoterapia*.

[9] FAO (1997). *Agriculture Food and Nutrition for Africa—A Resource Book for Teachers of Agriculture*.

[10] Rokaya MB, Munzbergova Z, Timsina B, Bhattarai KR (2012). *Rheum australe* D. Don: a review of its botany, ethnobotany, phytochemistry and pharmacology. *Journal of Ethnopharmacology*.

[11] Young PH, Hsu YJ, Yang WC, Awaad AS, Singh VK, Govil JN (2010). *Bidens pilosa* L. and its medicinal use. *Recent Progress in Medicinal Plants Drug Plant II*.

[12] Ge C (1990). Cytologic study of **Bidens bipinnata** L. *Zhongguo Zhong Yao Za Zhi*.

[13] Chiang LC, Chang JS, Chen CC, Ng LT, Lin CC (2003). Anti-herpes simplex virus activity of *Bidens pilosa* and **Houttuynia cordata**. *American Journal of Chinese Medicine*.

[14] Rybalchenko NP, Prykhodko VA, Nagorna SS (2010). In vitro antifungal activity of phenylheptatriyne from **Bidens cernua** L. against yeasts. *Fitoterapia*.

[15] Zhou Y, Yan XZ (1989). Experimental study of qi deficiency syndrome and **Codonopsis pillosulae** and **Astragalus injection** on the immune response in mice. *Zhong Xi Yi Jie He Za Zhi*.

[16] Redl K, Breu W, Davis B, Bauer R (1994). Anti-inflammatory active polyacetylenes from **Bidens campylotheca**. *Planta Medica*.

[17] Tan PV, Dimo T, Dongo E (2000). Effects of methanol, cyclohexane and methylene chlo ride extracts of *Bidens pilosa* on various gastric ulcer models in rats. *Journal of Ethnopharmacology*.

[18] Pereira RLC, Ibrahim T, Lucchetti L, Da Silva AJR, De Moraes VLG (1999). Immunosuppressive and anti-inflammatory effects of methanolic extract and the polyacetylene isolated from *Bidens pilosa* L. *Immunopharmacology*.

[19] Dimo T, Azay J, Tan PV (2001). Effects of the aqueous and methylene chloride extracts of *Bidens pilosa* leaf on fructose-hypertensive rats. *Journal of Ethnopharmacology*.

[20] Wiart C (2000). *Medicinal Plants of Southeast Asia*.

[21] Dharmananda S A Popular Remedy Ecapes Notice of Western Practitioners. http://www.itmonline.org/arts/bidens.htm.

[22] Lans C (2007). Comparison of plants used for skin and stomach problems in Trinidad and Tobago with Asian ethnomedicine. *Journal of Ethnobiology and Ethnomedicine*.

[23] Ayyanar M, Ignacimuthu S (2005). Traditional knowledge of Kani tribals in Kouthalai of Tirunelveli hills, Tamil Nadu, India. *Journal of Ethnopharmacology*.

[24] Cano JH, Volpato G (2004). Herbal mixtures in the traditional medicine of Eastern Cuba. *Journal of Ethnopharmacology*.

[25] Geissberger P, Sequin U (1991). Constituents of *Bidens pilosa* L.: do the components found so far explain the use of this plant in traditional medicine?. *Acta Tropica*.

[26] Noumi E, Houngue F, Lontsi D (1999). Traditional medicines in primary health care: plants used for the treatment of hypertension in Bafia, Cameroon. *Fitoterapia*.

[27] Silva FL, Fischer DCH, Tavares JF, Silva MS, De Athayde-Filho PF, Barbosa-Filho JM (2011). Compilation of secondary metabolites from *Bidens pilosa* L. *Molecules*.

[28] Wu LW, Chiang YM, Chuang HC (2007). A novel polyacetylene significantly inhibits angiogenesis and promotes apoptosis in human endothelial cells through activation of the CDK inhibitors and caspase-7. *Planta Medica*.

[29] Chang SL, Chiang YM, Chang CLT (2007). Flavonoids, centaurein and centaureidin, from *Bidens pilosa*, stimulate IFN-*γ* expression. *Journal of Ethnopharmacology*.

[30] Kumari P, Misra K, Sisodia BS (2009). A promising anticancer and antimalarial component from the leaves of *Bidens pilosa*. *Planta Medica*.

[31] Seelinger G, Merfort I, Wölfle U, Schempp CM (2008). Anti-carcinogenic effects of the flavonoid luteolin. *Molecules*.

[32] Seelinger G, Merfort I, Schempp CM (2008). Anti-oxidant, anti-inflammatory and anti-allergic activities of luteolin. *Planta Medica*.

[33] Yit CC, Das NP (1994). Cytotoxic effect of butein on human colon adenocarcinoma cell proliferation. *Cancer Letters*.

[34] Beutler JA, Hamel E, Vlietinck AJ (1998). Structure-activity requirements for flavone cytotoxicity and binding to tubulin. *Journal of Medicinal Chemistry*.

[35] Kviecinski MR, Felipe KB, Schoenfelder T (2008). Study of the antitumor potential of *Bidens pilosa* (Asteraceae) used in Brazilian folk medicine. *Journal of Ethnopharmacology*.

[36] Sundararajan P, Dey A, Smith A, Doss AG, Rajappan M, Natarajan S (2006). Studies of anticancer and antipyretic activity of *Bidens pilosa* whole plant. *African Health Sciences*.

[37] Horiuchi M, Seyama Y (2008). Improvement of the antiinflammatory and antiallergic activity of *Bidens pilosa* L. var. *radiata* SCHERFF treated with enzyme (Cellulosine). *Journal of Health Science*.

[38] Chiang YM, Lo CP, Chen YP (2005). Ethyl caffeate suppresses NF-*κ*B activation and its downstream inflammatory mediators, iNOS, COX-2, and PGE2 in vitro or in mouse skin. *British Journal of Pharmacology*.

[39] Kim JS, Lee HJ, Lee MH, Kim J, Jin C, Ryu JH (2006). Luteolin inhibits LPS-stimulated inducible nitric oxide synthase expression in BV-2 microglial cells. *Planta Medica*.

[40] Ruiz PA, Haller D (2006). Functional diversity of flavonoids in the inhibition of the proinflammatory NF-*κ*B, IRF, and Akt signaling pathways in murine intestinal epithelial cells. *Journal of Nutrition*.

[41] Xagorari A, Papapetropoulos A, Mauromatis A, Economou M, Fotsis T, Roussos C (2001). Luteolin inhibits an endotoxin-stimulated phosphorylation cascade and proinflammatory cytokine production in macrophages. *Journal of Pharmacology and Experimental Therapeutics*.

[42] Yoshida N, Kanekura T, Higashi Y, Kanzaki T (2006). *Bidens pilosa* suppresses interleukin-1*β*-induced cyclooxygenase-2 expression through the inhibition of mitogen activated protein kinases phosphorylation in normal human dermal fibroblasts. *Journal of Dermatology*.

[43] Hsu YJ, Lee TH, Chang CLT, Huang YT, Yang WC (2009). Anti-hyperglycemic effects and mechanism of *Bidens pilosa* water extract. *Journal of Ethnopharmacology*.

[44] Chang SL, Chang CLT, Chiang YM (2004). Polyacetylenic compounds and butanol fraction from *Bidens pilosa* can modulate the differentiation of helper T cells and prevent autoimmune diabetes in non-obese diabetic mice. *Planta Medica*.

[45] Ubillas RP, Mendez CD, Jolad SD (2000). Antihyperglycemic acetylenic glucosides from *Bidens pilosa*. *Planta Medica*.

[46] Chang CLT, Chang SL, Lee YM (2007). Cytopiloyne, a polyacetylenic glucoside, prevents type 1 diabetes in nonobese diabetic mice. *Journal of Immunology*.

[47] Chiang YM, Chuang DY, Wang SY, Kuo YH, Tsai PW, Shyur LF (2004). Metabolite profiling and chemopreventive bioactivity of plant extracts from *Bidens pilosa*. *Journal of Ethnopharmacology*.

[48] Muchuweti M, Mupure C, Ndhlala A, Murenje T, Benhura MAN (2007). Screening of antioxidant and radical scavenging activity of *Vigna ungiculata*, *Bidens pilosa* and *Cleome gynandra*. *American Journal of Food Technology*.

[49] Yuan LP, Chen FH, Ling L (2008). Protective effects of total flavonoids of *Bidens bipinnata* L. against carbon tetrachloride-induced liver fibrosis in rats. *Journal of Pharmacy and Pharmacology*.

[50] Chiang YM, Chang CLT, Chang SL, Yang WC, Shyur LF (2007). Cytopiloyne, a novel polyacetylenic glucoside from *Bidens pilosa*, functions as a T helper cell modulator. *Journal of Ethnopharmacology*.

[51] Tobinaga S, Sharma MK, Aalbersberg WGL (2009). Isolation and identification of a potent antimalarial and antibacterial polyacetylene from *Bidens pilosa*. *Planta Medica*.

[52] Chang SL, Yeh HH, Lin YS, Chiang YM, Wu TK, Yang WC (2007). The effect of centaurein on interferon-*γ* expression and *Listeria* infection in mice. *Toxicology and Applied Pharmacology*.

[53] Deba F, Xuan TD, Yasuda M, Tawata S (2008). Chemical composition and antioxidant, antibacterial and antifungal activities of the essential oils from *Bidens pilosa* Linn. var. *Radiata*. *Food Control*.

[54] Ashafa AOT, Afolayan AJ (2009). Screening the root extracts from *Biden pilosa* L. var. *radiata* (Asteraceae) for antimicrobial potentials. *Journal of Medicinal Plant Research*.

[55] Nguelefack TB, Dimo T, Nguelefack Mbuyo EP, Tan PV, Rakotonirina SV, Kamanyi A (2005). Relaxant effects of the neutral extract of the leaves of *Bidens pilosa* linn on isolated rat vascular smooth muscle. *Phytotherapy Research*.

[56] Abbas AK, Murphy KM, Sher A (1996). Functional diversity of helper T lymphocytes. *Nature*.

[57] Namukobe J, Kasenene JM, Kiremire BT (2011). Traditional plants used for medicinal purposes by local communities around the Northern sector of Kibale National Park, Uganda. *Journal of Ethnopharmacology*.

[58] Chen AH, Lin SR, Hong CH (1975). Phytochemical study on *Bidens pilosa* L. var. *minor*. *Huaxue*.

[59] Lee CK (2000). The low polar constituents from *Bidens pilosa* L. var. *minor* (blume) sherff. *Journal of the Chinese Chemical Society*.

[60] Zulueta MC, Tada M, Ragasa CY (1995). A diterpene from *Bidens pilosa*. *Phytochemistry*.

[61] Wang J, Yang H, Lin ZW, Sun HD (1997). Flavonoids from *Bidens pilosa* var. *radiata*. *Phytochemistry*.

[62] Zhao A, Zhao Q, Peng L A new chalcone glycoside from *Bidens pilosa*. *Acta Botanica Yunnanica*.

[63] Gao P, Wu Y, Cui S, Zou Y (2011). Synthesis and absolute configuration of the 7-phenylhepta-4, 6-diyne-1, 2-diol isolated from *Bidens pilosa*. *Synthesis*.

[64] Wang S, Yang B, Zhu D, He D, Wang L Active components of *Bidens pilosa* L. *Zhongcaoyao*.

[65] Bohlmann F, Burkhardt T, Zdero C (1973). *Naturally Occuring Acetylenes*.

[66] Sarg TM, Ateya AM, Farrag NM, Abbas FA (1991). Constituents and biological activity of *Bidens pilosa* L. grown in Egypt. *Acta Pharmaceutica Hungarica*.

[67] Valdés HAL, Rego HP *Bidens pilosa* Linné. *Revista Cubana de Plantas Medicinales*.

[68] Bohlmann F, Bornowski H, Kleine KM New polyynes from the tribe Heliantheae. *Chemische Berichte*.

[69] Wang R, Wu QX, Shi YP (2010). Polyacetylenes and flavonoids from the aerial parts of *Bidens pilosa*. *Planta Medica*.

[70] Wu LW, Chiang YM, Chuang HC (2004). Polyacetylenes function as anti-angiogenic agents. *Pharmaceutical Research*.

[71] Yang HL, Chen SC, Chang NW (2006). Protection from oxidative damage using *Bidens pilosa* extracts in normal human erythrocytes. *Food and Chemical Toxicology*.

[72] Wang HQ, Lu SJ, Li H, Yao ZH (2007). EDTA-enhanced phytoremediation of lead contaminated soil by **Bidens maximowicziana**. *Journal of Environmental Sciences*.

[73] Chang CLT, Kuo HK, Chang SL (2005). The distinct effects of a butanol fraction of *Bidens pilosa* plant extract on the development of Th1-mediated diabetes and Th2-mediated airway inflammation in mice. *Journal of Biomedical Science*.

[74] Alvarez L, Marquina S, Villarreal ML, Alonso D, Aranda E, Delgado G (1996). Bioactive polyacetylenes from *Bidens pilosa*. *Planta Medica*.

[75] Kusano G, Kusano A, Seyama Y (2004). *Novel Hypoglycemic and Anti-Inflammatory Polyacetylenic Compounds, Their Compositions, Bidens Plant Extract Fractions, and Compositions Containing the Plant or Fraction*.

[76] Brandão MGL, Krettli AU, Soares LSR, Nery CGC, Marinuzzi HC (1997). Antimalarial activity of extracts and fractions from *Bidens pilosa* and other *Bidens* species (Asteraceae) correlated with the presence of acetylene and flavonoid compounds. *Journal of Ethnopharmacology*.

[77] Krettli AU, Andrade-Neto VF, Brandão MDGL, Ferrari WMS (2001). The search for new anti-malarial drugs from plants used to treat fever and malaria or plants ramdomly selected: a review. *Memorias do Instituto Oswaldo Cruz*.

[78] Lee CK (2000). The low polar constituents from *Bidens pilosa* L. var. *minor* (blume) sherff. *Journal of the Chinese Chemical Society*.

[79] Wat CK, Biswas RK, Graham EA, Bohm L, Towers GHN, Waygood ER (1979). Ultraviolet-mediated cytotoxic activity of phenylheptatriyne from *Bidens pilosa* L. *Journal of Natural Products*.

[80] Sashida Y, Ogawa K, Kitada M, Karikome H, Mimaki Y, Shimomura H (1991). New aurone glucosides and new phenylpropanoid glucosides from *Bidens pilosa*. *Chemical and Pharmaceutical Bulletin*.

[81] Yuan LP, Chen FH, Ling L (2008). Protective effects of total flavonoids of *Bidens pilosa* L. (TFB) on animal liver injury and liver fibrosis. *Journal of Ethnopharmacology*.

[82] Hoffmann B, Hölzl J Acylated compounds from *Bidens pilosa*. *Planta Medica*.

[83] Hoffmann B, Hölzl J (1988). A methylated chalcone glucoside from *Bidens pilosa*. *Phytochemistry*.

[84] Hoffmann B, Hölzl J (1989). Chalcone glucosides from *Bidens pilosa*. *Phytochemistry*.

[85] Hoffmann B, Hölzl J New chalcones from *Bidens pilosa*. *Planta Medica*.

[86] Hoffmann B, Hölzl J (1988). Weitere acylierte chalkone aus *Bidens pilosa*. *Planta Medica*.

[87] Pham VV, K P VVT, Hoang VL, Phan VK Flavonoid compounds from the plant *Bidens pilosa* L., (Asteraceae). *Tap Chi Duoc Hoc*.

[88] Sarker SD, Bartholomew B, Nash RJ, Robinson N (2000). 5-O-methylhoslundin: an unusual flavonoid from *Bidens pilosa* (Asteraceae). *Biochemical Systematics and Ecology*.

[89] Kusano A, Seyama Y, Usami E (2003). Studies on the antioxidant active constituents of the dried powder from *Bidens pilosa* L. var. *radiata* Sch. *Natural Medicines*.

[90] Dolečková I, Rárová L, Grúz J Anti-proliferative and anti-angiogenic effects of flavone eupatorin, an active constituent of chloroform extract of *Orthosiphon stamineus* leaves. *Fitoterapia*.

[91] Bairwa K, Kumar R, Sharma RJ, Roy RK An updated review on *Bidens pilosa* L. *Der Pharma Chemica*.

[92] Xia Q, Liu Y, Li Y Determination of hyperoside in different parts and different species of Herba *Bidens* by RP-HPLC. *West China Journal of Pharmaceutical Sciences*.

[93] Brandão MGL, Nery CGC, Mamão MAS, Krettli AU (1998). Two methoxylated flavone glycosides from *Bidens pilosa*. *Phytochemistry*.

[94] Grombone-Guaratini MT, Silva-Brandão KL, Solferini VN, Semir J, Trigo JR (2005). Sesquiterpene and polyacetylene profile of the *Bidens pilosa* complex (Asteraceae: Heliantheae) from Southeast of Brazil. *Biochemical Systematics and Ecology*.

[95] Lin LL, Wu CY, Hsiu HC, Wang MT, Chuang H (1967). Studies on diabetes mellitus. I. The hypoglycemic activity of phytosterin on alloxan diabetic rats. *Taiwan Yi Xue Hui Za Zhi*.

[96] Benhura MAN, Chitsiku IC (1997). The extractable *β*-carotene content of Guku (*Bidens pilosa*) leaves after cooking, drying and storage. *International Journal of Food Science and Technology*.

[97] Deba F, Xuan TD, Yasuda M, Tawata S (2007). Herbicidal and fungicidal activities and identification of potential phytotoxins from *Bidens pilosa* L. var. *radiata* Scherff: research paper. *Weed Biology and Management*.

[98] Kumar JK, Sinha AK (2003). A new disubstituted acetylacetone from the leaves of *Bidens pilosa* Linn. *Natural Product Research*.

[99] Ogawa K, Sashida Y (1992). Caffeoyl derivatives of a sugar lactone and its hydroxy acid from the leaves of *Bidens pilosa*. *Phytochemistry*.

[100] Xia Q, Liu Y, Li Y Determination of gallic acid from different species and different medical parts of Herba *Bidens* by RP-HPLC. *West China Journal of Pharmaceutical Sciences*.

[101] Chang JS, Chiang LC, Chen CC, Liu LT, Wang KC, Lin CC (2001). Atileukemic activity of *Bidens pilosa* l. var. *minor* (blume) sherff and *Houttuynia cordata* thunb. *American Journal of Chinese Medicine*.

[102] Lee WJ, Wu LF, Chen WK, Wang CJ, Tseng TH (2006). Inhibitory effect of luteolin on hepatocyte growth factor/scatter factor-induced HepG2 cell invasion involving both MAPK/ERKs and PI3K-Akt pathways. *Chemico-Biological Interactions*.

[103] Beutler JA, Cardellina JH, Lin CM, Hamel E, Cragg GM, Boyd MR (1993). Centaureidin, a cytotoxic flavone from *Polymnia fruticosa*, inhibits tubulin polymerization. *Bioorganic and Medicinal Chemistry Letters*.

[104] Verma A, Su A, Golin AM, O’Marrah B, Amorosa JK (2001). The lateral view: a screening method for knee trauma. *Academic Radiology*.

[105] Corren J, Lemay M, Lin Y, Rozga L, Randolph RK (2008). Clinical and biochemical effects of a combination botanical product (ClearGuard) for allergy: a pilot randomized double-blind placebo-controlled trial. *Nutrition Journal*.

[106] Gachet MS, Lecaro JS, Kaiser M (2010). Assessment of anti-protozoal activity of plants traditionally used in Ecuador in the treatment of leishmaniasis. *Journal of Ethnopharmacology*.

[107] Yang WC, Ghiotto M, Barbarat B, Olive D (1999). The role of Tec protein-tyrosine kinase in T cell signaling. *The Journal of Biological Chemistry*.

[108] Tewtrakul S, Miyashiro H, Nakamura N (2003). HIV-1 integrase inhibitory substances from *Coleus parvifolius*. *Phytotherapy Research*.

[109] Dimo T, Nguelefack TB, Tan PV (2003). Possible mechanisms of action of the neutral extract from *Bidens pilosa* L. leaves on the cardiovascular system of anaesthetized rats. *Phytotherapy Research*.

[110] Li TSC (2002). *Chinese and Related North American Herbs*.

[111] Wu H, Chen H, Hua X, Shi Z, Zhang L, Chen J (1997). Clinical therapeutic effect of drug-separated moxibustion on chronic diarrhea and its immunologic mechanisms. *Journal of Traditional Chinese Medicine*.

[112] Wright SW, Harris RR, Kerr JS (1992). Synthesis, chemical, and biological properties of vinylogous hydroxamic acids: dual inhibitors of 5-lipoxygenase and IL-1 biosynthesis. *Journal of Medicinal Chemistry*.

[113] Almirón WR, Brewer ME (1996). Classification of immature stage habitats of *Culicidae* (Diptera) collected in Cordoba, Argentina. *Memorias do Instituto Oswaldo Cruz*.

[114] Wang NL, Wang J, Yao XS, Kitanaka S (2007). Two new monoterpene glycosides and a new (+)-jasmololone glucoside from *Bidens parviflora* Willd. *Journal of Asian Natural Products Research*.

[115] Champagnat P (1951). Role of the terminal bud in the action exercised by the cotyledon of *Bidens pilosus* L. var. *radiatus* on its axillary bud. *Comptes Rendus des Séances et Mémoires de la Société de Biologie*.

[116] Nielsen SF, Christensen SB, Cruciani G, Kharazmi A, Liljefors T (1998). Antileishmaniai chalcones: statistical design, synthesis, and three-dimensional quantitative structure-activity relationship analysis. *Journal of Medicinal Chemistry*.

[117] Hwang YC, Chu JJH, Yang PL, Chen W, Yates MV (2008). Rapid identification of inhibitors that interfere with poliovirus replication using a cell-based assay. *Antiviral Research*.

[118] Andrade-Neto VF, Brandão MGL, Oliveira FQ (2004). Antimalarial activity of *Bidens pilosa* L. (Asteraceae) ethanol extracts from wild plants collected in various localities or plants cultivated in humus soil. *Phytotherapy Research*.

[119] Marles RJ, Farnsworth NR Anti-diabetic plants and their active constituents. *Phytomedicine*.

[120] Habeck M (2003). Diabetes treatments get sweet help from nature. *Nature Medicine*.

[121] Lin HW, Han GY, Liao SX (1994). Studies on the active constituents of the Chinese traditional medicine *Polygonatum odoratum* (Mill.) Druce. *Acta Pharmaceutica Sinica*.

[122] Chang CLT, Chen YC, Chen HM, Yang NS, Yang WC (2013). Natural cures for type 1 diabetes: a review of phytochemicals, biological actions, and clinical potential. *Current Medicinal Chemistry*.

[123] Dey L, Attele AS, Yuan CS (2002). Alternative therapies for type 2 diabetes. *Alternative Medicine Review*.

[124] Chang CLT, Liu HY, Kuo TF (2013). Anti-diabetic effect and mode of action of cytopiloyne. *Evidence-Based Complementary and Alternative Medicine*.

[125] Khan MR, Kihara M, Omoloso AD (2001). Anti-microbial activity of *Bidens pilosa*, *Bischofia javanica, Elmerillia papuana* and *Sigesbekia orientalis*. *Fitoterapia*.

[126] Dimo T, Nguelefack TB, Kamtchouing P, Dongo É, Rakotonirina A, Rakotonirina SV (1999). Hypotensive effects of methanol extract from *Bidens pilosa* Linn on hypertensive rats. *Comptes Rendus de l’Academie des Sciences. Serie III*.

[127] Hassan AK, Deogratius O, Nyafuono JF, Francis O, Engeu OP (2011). Wound healing potential of the ethanolic extracts of *Bidens pilosa* and *Ocimum suave*. *African Journal of Pharmacy and Pharmacology*.

[128] Hoffmann B, Hölzl J (1989). Chalcone glucosides from *Bidens pilosa*. *Phytochemistry*.

[129] Hoffmann B, Holzl J New chalcones from *Bidens pilosa*. *Planta Medica*.

[130] Kandaswami C, Middleton E (1994). Free radical scavenging and antioxidant activity of plant flavonoids. *Advances in Experimental Medicine and Biology*.

[131] Yamamoto K, Kakegawa H, Ueda H (1992). Gastric cytoprotective anti-ulcerogenic actions of hydroxychalcones in rats. *Planta Medica*.

[132] Ezeonwumelu JOC, Julius AK, Muhoho CN Biochemical and histological studies of aqueous extract of *Bidens pilosa* leaves from Ugandan Rift valley in rats. *British Journal of Pharmacology and Toxicology*.

[133] Frida L, Rakotonirina S, Rakotonirina A, Savineau JP (2008). In vivo and in vitro effects of *Bidens pilosa* L. (Asteraceae) leaf aqueous and ethanol extracts on primed-oestrogenized rat uterine muscle. *African Journal of Traditional, Complementary and Alternative Medicines*.

